# Structure-Guided Development of Bivalent Aptamers Blocking SARS-CoV-2 Infection

**DOI:** 10.3390/molecules28124645

**Published:** 2023-06-08

**Authors:** Md Shafiqur Rahman, Min Jung Han, Sang Won Kim, Seong Mu Kang, Bo Ri Kim, Heesun Kim, Chang Jun Lee, Jung Eun Noh, Hanseong Kim, Jie-Oh Lee, Sung Key Jang

**Affiliations:** 1 Department of Life Sciences, POSTECH Biotech Center, Pohang University of Science and Technology, 77 Cheongam-ro, Nam-gu, Pohang-si 37673, Republic of Korea; shafiqmb@postech.ac.kr (M.S.R.); hmj@postech.ac.kr (M.J.H.); ksw5758@postech.ac.kr (S.W.K.);; 2 Division of Integrative Bioscience & Biotechnology, POSTECH Biotech Center, Pohang University of Science and Technology, Nam-gu, Pohang-si 37673, Republic of Korea

**Keywords:** SARS-CoV-2, aptamer–RBD complex, cryo-EM structure, Nap-dU, neutralization, viro-SELEX

## Abstract

Severe acute respiratory syndrome coronavirus 2 (SARS-CoV-2) has caused devastation to human society through its high virulence, infectivity, and genomic mutations, which reduced the efficacy of vaccines. Here, we report the development of aptamers that effectively interfere with SARS-CoV-2 infection by targeting its spike protein, which plays a pivotal role in host cell entry of the virus through interaction with the viral receptor angiotensin-converting enzyme 2 (ACE2). To develop highly effective aptamers and to understand their mechanism in inhibiting viral infection, we determined the three-dimensional (3D) structures of aptamer/receptor-binding domain (RBD) complexes using cryogenic electron microscopy (cryo-EM). Moreover, we developed bivalent aptamers targeting two distinct regions of the RBD in the spike protein that directly interact with ACE2. One aptamer interferes with the binding of ACE2 by blocking the ACE2-binding site in RBD, and the other aptamer allosterically inhibits ACE2 by binding to a distinct face of RBD. Using the 3D structures of aptamer–RBD complexes, we minimized and optimized these aptamers. By combining the optimized aptamers, we developed a bivalent aptamer that showed a stronger inhibitory effect on virus infection than the component aptamers. This study confirms that the structure-based aptamer-design approach has a high potential in developing antiviral drugs against SARS-CoV-2 and other viruses.

## 1. Introduction

In December 2019, the novel coronavirus, severe acute respiratory syndrome coronavirus 2 (SARS-CoV-2), was identified for the first time in Wuhan, China [1,2], and afterward caused a global pandemic. SARS-CoV-2 caused the coronavirus disease 2019 (COVID-19). Over 765 million people were infected by SARS-CoV-2, and more than 6.9 million deaths attributed to COVID-19 were confirmed up to 10 May 2023 [3]. SARS-CoV-2’s spike protein is essential for the virus to enter the host cell through interaction with the viral receptor protein, angiotensin-converting enzyme 2 (ACE2) [4,5,6]. The spike protein consists of S1 and S2 subunits, and the S1 subunit contains an N-terminal domain (NTD) and a receptor-binding domain (RBD) [4,7]. The receptor-binding motif (RBM) of RBD is responsible for interaction with the helix in the N-terminal peptidase domain of ACE2 [5]. Owing to its essential role in virus infection, the trimeric spike protein of SARS-CoV-2 has become the major target for vaccine development, neutralizing antibodies (nAbs), drugs, and SARS-CoV-2 diagnosis [5,8].

Several vaccines have been developed to prevent infection of SARS-CoV-2, and they play a key role in preventing COVID-19 and/or reducing the severity of COVID-19 [9,10,11,12]. Moreover, a few drugs that have been found to be effective against COVID-19 originated from antiviral drug candidates for other viruses. For instance, Paxlovid, a protease inhibitor, and Molnupiravir, an RNA-dependent RNA polymerase inhibitor, are given to COVID-19 patients, even though their efficacy is limited [13,14]. Alternatively, nAbs targeting the spike have been used to treat patients with COVID-19 [15]. However, potential adverse effects of antibodies, such as antibody-dependent enhancement (ADE), represent a concern for antibody-based therapy [16,17]. The major concern for COVID-19 is the evolution of SARS-CoV-2. Numerous variants of SARS-CoV-2 have been discovered that escape from the immune response stimulated by vaccines and antiviral drugs [12,18,19,20]. Therefore, it is essential to conduct additional research for the development of better vaccines and antiviral medications to combat SARS-CoV-2 variants.

Aptamers are short, single-stranded oligonucleotides (DNA or RNA) that exhibit high affinity and specificity for a range of target molecules. Systematic evolution of ligands by exponential enrichment (SELEX) is a method used to generate aptamers [21]. Compared to antibodies, aptamers have a number of benefits. For example, aptamers can be produced chemically, are produced at low cost, exhibit low batch variability, can be readily modified, are smaller, and provoke a very low immune response [22]. Additionally, the use of modified nucleotide bases in lieu of conventional nucleotide bases has been shown to increase affinity and stability [23,24,25,26]. Therefore, it is anticipated that in the near future, aptamers will be used often in diagnostic and therapeutic applications. In the context of SARS-CoV-2, several studies have reported the development of aptamers against RBD of SARS-CoV-2 for diagnostic and therapeutic purposes [27,28,29,30,31]. Notably, one study developed an inhibitory aptamer independent of RBD [32]. Insights for the minimization and optimization of the aptamer might be gained from the structural information of an aptamer–protein complex, which would provide an understanding of the mechanism behind the interaction between the aptamer and its target protein [33]. However, to date, there is no structural analysis of an aptamer–SARS-CoV-2 spike complex showing the inhibition of SARS-CoV-2 infection, except for one aptamer binding to NTD of SARS-CoV-2 used for virus detection [34].

Here, we report the generation of aptamers that bind strongly to the spike protein of SARS-CoV-2 using viro-SELEX combined with conventional SELEX technology [35,36]. We determined the structure of aptamers bound to the RBD of SARS-CoV-2 using cryogenic electron microscopes (cryo-EM). Through the analysis of the aptamer/RBD complex structure, we revealed the molecular bases of the binding of aptamers to the target molecule. The inhibitory effects of aptamers on SARS-CoV-2 infection were investigated using pseudoviruses with envelope proteins containing the spike proteins of SARS-CoV-2 [31,37], mimicking the entry process of SARS-CoV-2. Two aptamers, namely AM032-0 and AM047-0, inhibited the infection of pseudoviruses. Furthermore, we optimized the aptamers to have minimum sizes and stable configurations based on the three-dimensional structure of the aptamer/RBD complex. Finally, we generated bivalent aptamers that comprised two optimized aptamers, AM032-4 and AM047-6, connected together by flexible linkers. A bivalent aptamer composed of the optimized aptamers showed a very strong inhibitory effect on the pseudovirus infection.

## 2. Results

### 2.1. Selection and Characterization of Aptamers Binding to Spike Proteins of SARS-CoV-2

To develop aptamers that interact strongly and specifically with the spike proteins of SARS-CoV-2, we performed viro-SELEX and conventional protein-SELEX [35,36] alternately using a modified nucleotide, i.e., 5-[N-(1-naphthylmethyl) carboxamide]-2′-deoxy uridine (Nap-dU), instead of the natural nucleotide deoxythymidine [23]. We used Nap-dU instead of deoxythymidine to increase the probability of acquiring aptamers with high affinities to the target molecule [23,24,25,26]. In the SELEX process, we used a purified trimeric spike protein of wild-type (Wuhan strain) SARS-CoV-2 (Supplementary Figure S1a) for protein-SELEX and a modified baculovirus (surrogate virus) containing wild-type spike proteins (Supplementary Figure S1d) on the viral envelope for viro-SELEX [35,36] to enrich aptamers binding to the natural form of spikes present on SARS-CoV-2; this was in accordance with the scheme presented in Supplementary Figure S2a. Moreover, purified trimeric spike proteins of other variants (D614G, alpha, beta, and delta) of SARS-CoV-2 were used to investigate the binding of aptamers to the variants (Supplementary Figure S1a). The trimeric nature of spike proteins was further confirmed by size exclusion chromatography (SEC) and cryo-EM (Supplementary Figure S1e,h).

**Figure 1:**
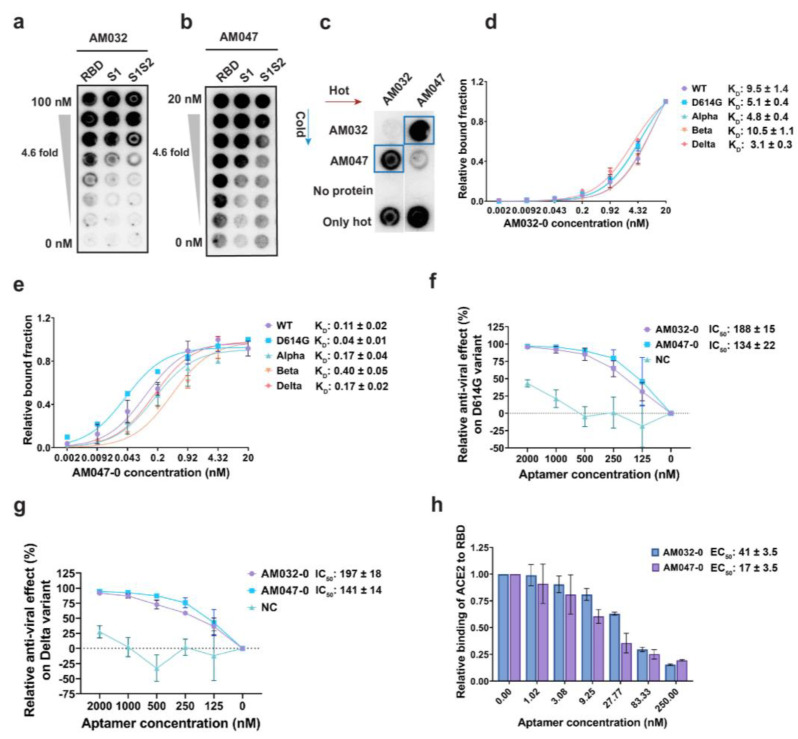
Development and characterization of aptamers. (**a**,**b**) Filter binding assays showing the binding of aptamers AM032 and AM047 to the various parts of a SARS-CoV-2 spike protein. (**c**) Protein-binding competition assay against S1S2 protein. [P^32^]-labeled (hot) aptamers and unlabeled (cold) aptamers were used as probes and competitors, respectively. (**d**,**e**) Binding affinities of AM032-0 (**d**) and AM047-0 (**e**) to the spike proteins of SARS-CoV-2 variants. The binding affinities were determined by filter binding assays (see Section 4). (**f**,**g**) Inhibitory effects of aptamers on the infection of SARS-CoV-2 pseudoviruses containing spike proteins of D614G (**f**) and delta (**g**) variants. (**h**) Inhibitory effects of aptamers on the interaction between ACE2 and RBD. Enzyme-linked immunosorbent assays (ELISAs) were performed to monitor the inhibitory effects. NC, negative control. The values presented for K_D_, IC_50_, and EC_50_ were measured in nM.

After carrying out SELEX, the protein-binding affinities of DNA pools from the 9th–12th rounds of SELEX were examined using filter binding assay [35]. For example, the DNA pool from 10th round showed strong binding affinities to various parts of the wild-type spike (WT-S) protein without showing binding affinity to spike proteins of other human betacoronaviruses HCoV HKU1 and HCoV OC43, which cause common cold (Supplementary Figure S2b). Among the DNA pools evaluated, we selected the DNA pool from the 11th round of SELEX, which showed the strongest binding affinity to the S1 subunit and WT-S proteins (K_D_ of ~0.45 nM and ~0.25 nM, respectively; Supplementary Figure S2c), for cloning plus sequencing by Sanger method and for direct sequencing by the next-generation sequencing (NGS) method. It is noteworthy to mention that many aptamers with various affinities to the spike protein were identified (Supplementary Figure S2f). Among the aptamers, we selected the two aptamers AM032 and AM047, which strongly bound to the RBD of the wild-type SARS-CoV-2 spike, for further analyses (Figure 1a,b). In addition, as shown in Figure 1c, aptamers AM032 and AM047 did not compete for binding to the RBD, indicating their different binding sites in the spike protein. Next, we synthesized derivatives of AM032 and AM047 with serial truncations from both 5′ and 3′ ends and measured their binding affinities to spike proteins. We selected an 80-mer derivative of AM032 and a 52-mer derivative of AM047 (designated as AM032-0 and AM0470, respectively), which had minimum sizes without losing binding affinities, for further studies. Both aptamers, i.e., AM032-0 and AM047-0, showed strong binding affinities to the spike proteins of SARS-CoV-2 variants in the K_D_ ranges of 3 nM–10.5 nM and 0.04 nM–0.4 nM, respectively (Figure 1d,e; Supplementary Figure S2d,e; and Table S1).

### 2.2. Aptamers AM032-0 and AM047-0 Block SARS-CoV-2 Infection through Inhibition of the Interaction between RBD and ACE2

Since aptamers AM032-0 and AM047-0 bind to the RBD of spike protein, they have the potential to inhibit SARS-CoV-2 infection by interfering with the interaction between spike protein and ACE2 proteins. We investigated their effects on the infectivity of pseudoviruses, which mimic the entry step of SARS-CoV-2 into a host cell as mediated by the spike proteins of SARS-CoV-2 on the surface of the pseudoviruses. Pseudoviruses containing the spike proteins of either D614G or delta variant and HEK293T cells expressing human ACE2 were used as the pseudoviruses and host cells, respectively. Firefly luciferase activity from the reporter gene integrated into the RNA of a pseudovirus was monitored to determine its infectivity. Pseudoviruses were cultured for 48 h in the presence of serially diluted aptamers, and the effects of aptamers on the infectivity of pseudoviruses were monitored by measuring firefly luciferase activities in the cell extracts. Both aptamers AM032-0 and AM047-0 inhibited infection of the pseudoviruses containing the spike proteins of D614G or delta variant in dose-dependent manners (Figure 1f,g). The half-maximal inhibitory concentrations (IC_50_s) of AM032-0 and AM047-0 against the pseudovirus with D614G spike were 188 nM and 134 nM, respectively (Figure 1f). Additionally, the IC_50_s of AM032-0 and AM047-0 against the pseudovirus with the delta spike were 197 nM and 141 nM, respectively (Figure 1g). The results indicate that both aptamers AM032-0 and AM047-0 interfered with the infection of SARS-CoV-2 variants and that the aptamer AM047-0, which had higher affinity to the spike protein than aptamer AM032-0, also had a higher inhibitory effect on SARS-CoV-2 infection. To understand the underlying mechanism of the interference of SARS-CoV-2 infection by the aptamers AM032-0 and AM047-0, we examined whether they inhibited the interaction between the spike and ACE2 proteins. Therefore, we performed enzyme-linked immunosorbent assays (ELISAs) measuring the interaction between ACE2 and the RBD of wild-type spike in the presence or absence of aptamers (Figure 1h). Both aptamers AM032-0 and AM047-0 strongly inhibited the interaction between ACE2 and the wild-type RBD. The half-maximal effective concentrations (EC_50_s) of aptamers AM032-0 and AM047-0 were 41.0 nM and 17.4 nM, respectively (Figure 1h). Again, the binding affinities of aptamers were well correlated with the interfering actions of aptamers in the interaction between ACE2 and RBD. Taken together, these findings conclude that aptamers AM032-0 and AM047-0 inhibit SARS-CoV-2 infection by interfering with the interaction between the spike and ACE2 proteins.

### 2.3. Determination of the Three-Dimensional Structure of RBD–Aptamer Complex by Cryo-EM

**Figure 2:**
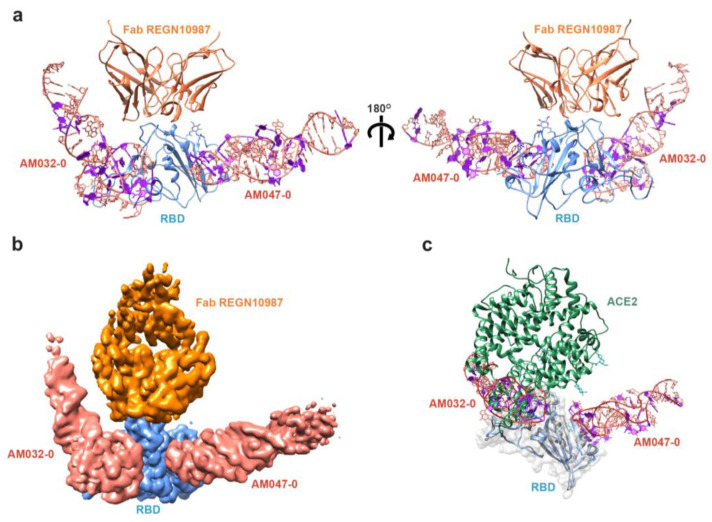
Cryo-EM structure of the RBD–Fab–AM032-0–AM047-0 complex. (**a**) Structure of the RBD–Fab–AM032-0–AM047-0 complex. Front view (left) and back view (right). (**b**) Cryo-EM density map of the protein–aptamer complex. (**c**) Structure of the RBD–aptamer complex superimposed with that of the RBD–ACE2 complex (PDB ID: 6M0J). Nap-dU residues of the aptamers are shown in purple.

To understand the detailed molecular mechanism of the aptamer binding to the spike protein and the inhibition of SARS-CoV-2 infection, we determined the three-dimensional structure of the RBD–aptamer complex using cryo-EM. We used a previously published Fab domain of an antibody, namely REGN10987 [38], against the RBD of the spike to increase the overall size of the RBD–aptamer complex and make it suitable for cryo-EM analysis (Supplementary Figure S3a). Both aptamers AM032-0 and AM047-0 were applied to the RBD–antibody complex to acquire a complex containing the aptamers: RBD–Fab–AM032-0–AM047-0. From 21,340 movie frames and from post-processing the cryo-EM movies in cryoSPARC [39], we obtained the cryo-EM map of the protein–aptamer complex at an overall resolution of 3.32 Å (Supplementary Figure S3b–f and Table S2). The initial atomic model for the protein part was constructed by fitting the previously published structures of RBD (PDB code 7KGJ) [40] and Fab (PDB code 6XDG) [38] to the map. The aptamer structures were built de novo using Coot [41] (see Materials and Methods for detail). The cryo-EM map corresponding to the constant region of Fab and the 5′ and 3′ end areas of the aptamer AM032-0 were not clearly visible, presumably due to high structural flexibility, and were therefore omitted from the refined structure (Figure 2a,b and Supplementary Figure S3g). The RBD structure of the complex is superimposable with a root mean square (r.m.s.) deviation of 1.41 Å and 1.48 Å (193 Cα pairs) with PDB IDs 6M0J [5] and 7KGJ [40], respectively, demonstrating that no significant conformational changes in the RBD molecule were induced by the aptamer binding. The complex structure illustrates that the aptamers AM032-0 and AM047-0 bind to two different sites in RBD (Figure 2a,b). AM032-0 stands astride the ACE2-binding surface of RBD, spanning residues from G476 to Y505 and distant residues R403 and F456. AM047-0 binds to an area composed of residues from Y365 to N388 that is distal from the ACE2-binding site (Figure 2c). The bound aptamers adopt regular double-helical structures at the areas close to their 5′ and 3′ ends but form complicated loop structures near the RBD–aptamer interface areas (Figure 2a).

**Figure 3:**
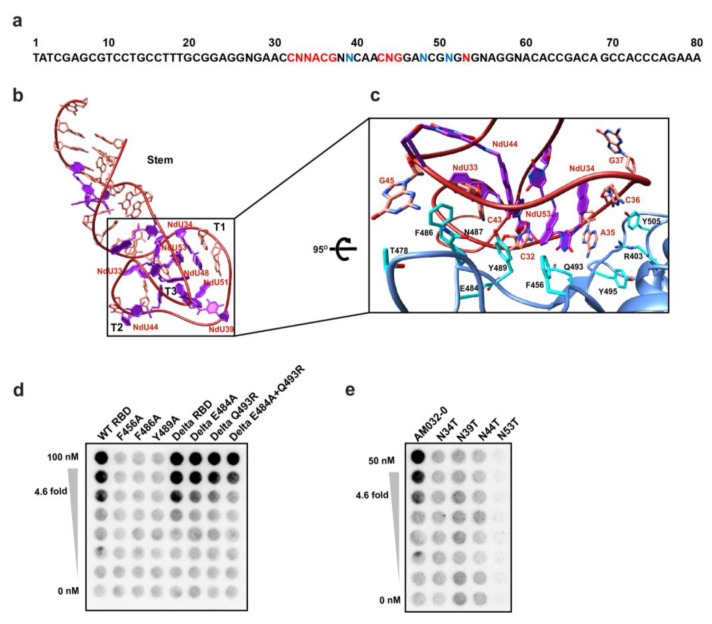
Structure of AM032-0 bound to the RBD of SARS-CoV-2. (**a**) Sequence of the aptamer AM032-0. The bases required for RBD binding and aptamer folding are written in red and blue, respectively. (**b**) The overall structure of aptamer AM032-0. For clarity, some nucleotides are not shown. Ndu represents Nap-dU. (**c**) Close-up view of the binding interface between AM032-0 and RBD. AM032-0 and RBD are colored in red and light blue, respectively. Nap-dU nucleotides (NdU) and amino acids critical for binding are shown in purple and cyan, respectively. (**d**) Effects of mutations in RBD on binding to aptamers. Mutations in RBDs of wild type (lanes 2–4) and delta variant (lanes 6–8) were introduced, and the effects of the mutations on binding to aptamer AM032-0 were monitored by filter binding assay. (**e**) Effects of nucleotide substitution of aptamer AM032-0 on binding to wild-type RBD. N, Nap-dU = 5-[N-(1-naphthylmethyl) carboxamide]-2′-deoxyuridine; A, 2′-deoxyadenosine; T, 2′-deoxythymidine; G, 2′-deoxyguanosine; C, 2′-deoxycytidine.

Aptamer AM032-0 is composed of 80 nucleotides with 11 modified Nap-dU nucleotides (Figure 3a). The three-dimensional structure of AM032-0 has one stem and three turns, i.e., T1, T2, and T3 (Figure 3b; see Supplementary Note for structural details). This unusual three-turn structure is stabilized by a hydrophobic core composed of seven Nap-dU residues at positions 33, 34, 39, 44, 48, 51, and 53 (Figure 3b,c). Mutagenesis of some of these nucleotides individually with deoxythymidine abolishes the binding of the aptamer to RBD (Figure 3e). Among the three turns, the T1 plays the main role in RBD binding. Bases 32, 34, 35, 36, and 43 make intimate polar interactions with side chains of N487, Q493, R403, and Y505 (Figure 3c and Supplementary Table S3). The aromatic amino acids F456, F486, and Y489 of RBD make hydrophobic interactions with C32, Nap-dU33, Nap-dU34, C43, Nap-dU44, G45, and Nap-dU53 (Figure 3c and Supplementary Table S3). Mutations of these aromatic residues disrupt aptamer binding (Figure 3d).

**Figure 4:**
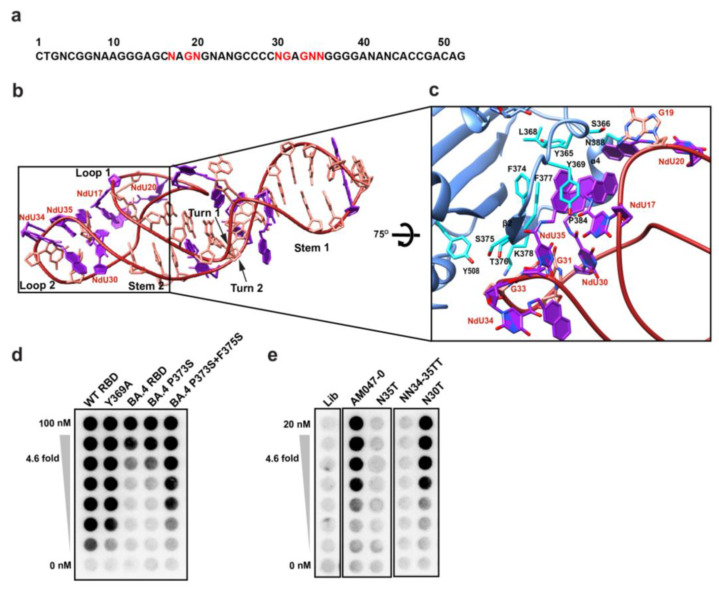
Structure of AM047-0 bound to the RBD of SARS-CoV-2. (**a**) Sequence of the aptamer AM047-0. The bases required for RBD binding are written in red. (**b**) The overall structure of AM047-0. (**c**) Close-up view of the binding interface between AM047-0 (red) and RBD (light blue). Nap-dUs are shown in purple. (**d**) Effects of mutations in RBD on binding to aptamers. Mutations in RBDs of wild type (lane 2) and BA.4/5 variant (lanes 4–5; BA.4/5 denoted as BA.4) were introduced, and the effects of the mutations on binding to aptamer AM047-0 were monitored by filter binding assay. (**e**) Effects of nucleotide substitution of aptamer AM047-0 aptamer on binding to wild-type RBD. N, Nap-dU = 5-[N-(1-naphthylmethyl) carboxamide]-2′-deoxyuridine; A, 2′-deoxyadenosine; T, 2′-deoxythymidine; G, 2′-deoxyguanosine; C, 2′-deoxycytidine; Lib, aptamer library.

The aptamer AM047-0 comprises 52 nucleotides with 11 Nap-modified nucleotides (Figure 4a). The refined structure of AM047-0 in complex with RBD has two stems, S1 and S2; two turns, T1 and T2; and two loops, L1 and L2 (Figure 4b; see Supplementary Note for structural details). The L1 and L2 loops of AM047-0 are responsible for binding to RBD protein. The aptamer-binding site consists of beta-strand β2, alpha helix α4, and a loop preceding the β2 strand of RBD (Figure 4c). Both hydrophobic and polar interactions mediated by several residues are involved in the interaction with RBD (Figure 4c and Supplementary Table S3). The Nap-dU residues at positions 30 and 35 of the L2 loop and positions 17 and 20 of the L1 loop form a hydrophobic surface in the aptamer that is critical in the interaction with the hydrophobic surface formed by Y365, L368, Y369, F374, F377, P384, and L387 residues of the spike protein (Figure 4c and Supplementary Table S3). This core hydrophobic interaction is supported by hydrophilic interactions in the periphery. The G19 residue of the L1 loop makes a hydrogen bond with S366 residue of RBD. The G31 and G33 residues of the L2 loop make a polar interaction with T376 and K378 residues of the spike (Figure 4c and Supplementary Table S3). Consistent with these observations, the replacement of Nap-dU35 with dT abolished the binding of aptamer AM047-0 with RBD (Figure 4e), suggesting its central role in binding and stabilizing the interface structure of AM047-0. This hypothesis was also supported by the binding assay showing that the reverting of the amino acid mutations in omicron BA.4/5 (P373 and F375), which binds weakly to AM047-0, to the wild-type (S373 and S375) restores the binding to AM047-0 (Figure 4d and Supplementary Table S4).

### 2.4. Aptamers AM032-0 and AM047-0 Block the Association of ACE2 to RBD through Distinct Mechanisms

As shown in Figure 1f–h, both AM032-0 and AM047-0 aptamers compete with ACE2 for binding to the spike protein and inhibit the infection of SARS-CoV-2 pseudovirus. The inhibition of the ACE2–RBD interaction by AM032-0 is easily comprehensible since ACE2 and AM032-0 occupy an overlapped region on RBD, as shown in Figure 2c. Additionally, several amino acid residues in RBD (F456, F486, Y489, and Q493) participate in the interactions with both ACE2 [5] and AM032-0 (Figure 2c and Figure 3c). Therefore, ACE2 and AM032-0 cannot bind to the spike protein at the same time since the binding of one molecule (either ACE2 or AM032-0) would not allow the binding of the other molecule to the spike (Figure 1h). However, aptamer AM047-0 binds to RBD at a distinct region from the ACE2-binding site, and AM047-0 and ACE2 may not directly bump into each other even if both of the molecules are associated with RBD, as shown in Figure 2c. A possible explanation for the inhibitory effect of AM047-0 is described in Section 3.

### 2.5. Optimization of Aptamers AM032-0 and AM047-0

Optimization of aptamers (an increase of binding affinity, minimization of size, minimization of modified nucleotide Nap-dU, reduction of susceptibility to nucleases, and stabilization of structure) is required for use in diagnostic or therapeutic purposes. Through the structural analysis of the aptamers AM032-0 and AM047-0, we recognized that the 5′ and 3′ ends of the aptamers form partially double-stranded configurations that stabilize the overall structures of the aptamers (Figure 2a). On the other hand, the central parts of the aptamers AM032-0 and AM047-0 form complex structures participating in the protein bindings (Figure 3b,c and Figure 4b,c). We designed several aptamers to confirm the structures and to optimize aptamers (Supplementary Figure S4a,b). In the optimization process, we maintained the central regions of the aptamers in order to not disturb the structures required for protein binding. On the other hand, we removed the peripheral regions as much as possible to minimize the aptamer size and changed the sequences to develop strong stem structures with many G-C base pairings (Supplementary Figure S4a,b) to stabilize the aptamer structures [31,42].

**Figure 5:**
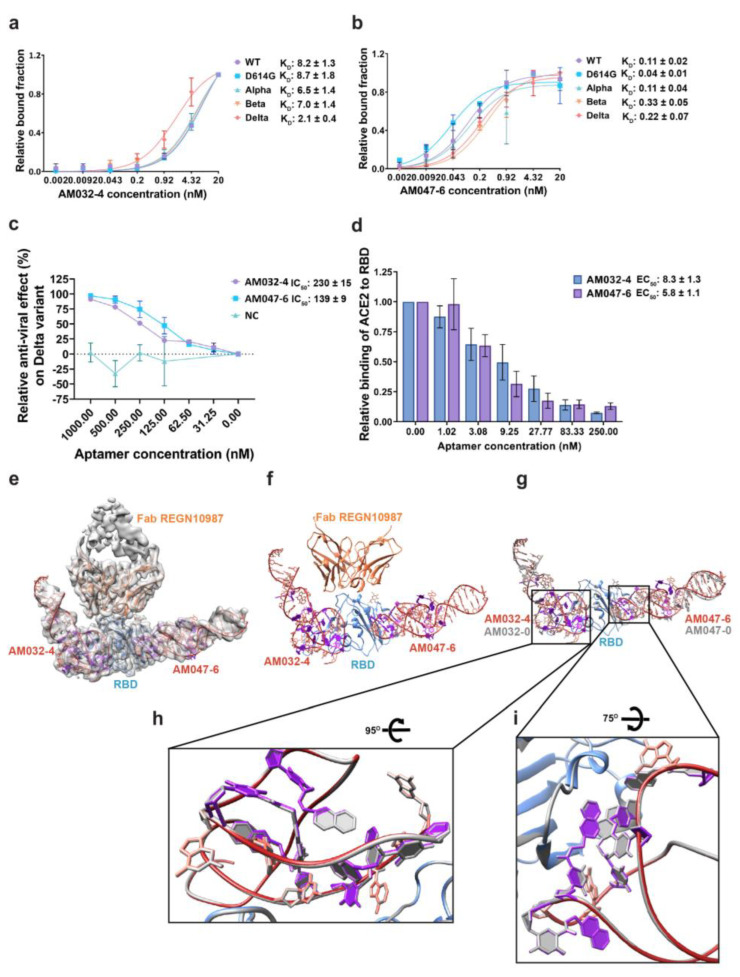
Characterization of aptamers AM032-4 and AM047-6 and cryo-EM structure of RBD–Fab–AM032-4–AM047-6 complex. (**a**,**b**) Binding affinities to the spike proteins of SARS-CoV-2 variants. (**c**) Inhibitory effects of aptamers on the infection of SARS-CoV-2 pseudovirus containing the spike protein of the delta variant. (**d**) Inhibitory effects of aptamers on the interaction between ACE2 and RBD. ELISAs were performed to monitor the inhibitory effects. (**e**) Cryo-EM density map of the protein–aptamer complex (front view) is superimposed with the refined model. (**f**) Structure of the RBD–Fab–AM032-4–AM047-6 complex. (**g**) The structure of the RBD–Fab–AM032-4–AM047-6 complex superimposed with that of the RBD–Fab–AM032-0–AM047-0 complex (gray). Nap-dUs of AM032-4 and AM047-6 are shown in purple. (**h**,**i**) Structures of the binding interfaces of AM032-4 (**h**) and AM047-6 (**i**) are superimposed with that of the RBD-Fab-AM032-0-AM047-0 complex (gray). NC, negative control. The values presented for K_D_, IC_50_, and EC_50_ were measured in nM.

Various oligonucleotides related to AM032-0 and AM047-0 were synthesized, and their binding affinities to wild-type RBD were monitored by filter binding assays (Supplementary Figure S4c,d). Aptamers AM032-4 and AM047-6 (Supplementary Figures S4a,b and S5h,i) were the finally optimized aptamers that had strong binding affinities to the spike proteins of SARS-CoV-2 variants (Figure 5a,b; Supplementary Figure S4e,f; and Supplementary Table S1). To evaluate the antiviral activities of the optimized aptamers, we analyzed the effects of AM032-4 and AM047-6 on the RBD–ACE2 interaction and their inhibitory effects on the infection of the SARS-CoV-2 delta pseudovirus (Figure 5c,d). AM032-4 and AM047-6 inhibited RBD–ACE2 interaction with EC_50_ values of 8.3 nM and 5.8 nM, respectively. Notably, AM032-4 and AM047-6 demonstrated approximately five and three times stronger inhibitory effects compared with the original aptamers, AM032-0 and AM047-0 (Figure 1h and Figure 5d). However, the inhibitory effects of AM032-4 and AM047-6 on pseudovirus infection were similar to those of AM032-0 and AM047-0 (Figure 1g and Figure 5c).

### 2.6. Structure of RBD–Fab–AM032-4–AM047-6 Complex

We determined the structures of the optimized aptamers complexed with RBD associated with Fab since the optimized aptamers were likely to show higher resolution due to their compact structures around the termini with strengthened double-stranded stems. Indeed, we obtained the cryo-EM map of the protein–aptamer complex at a resolution of 3.43 Å, with an improved resolution in the binding interfaces and less flexibility at the termini of aptamers (Figure 5e–g; Supplementary Figure S5a–g; and Table S2). As expected, these aptamers have similar structures at the binding interfaces compared to AM032-0 and AM047-0 (Figure 5h,i). Major differences were observed at the 5′ and 3′ ends, where truncations and changes in bases were newly introduced in the optimized aptamers (Figure 5g).

### 2.7. Generation of a Bivalent Aptamer That Strongly Inhibits SARS-CoV-2 Pseudovirus Infection

**Figure 6:**
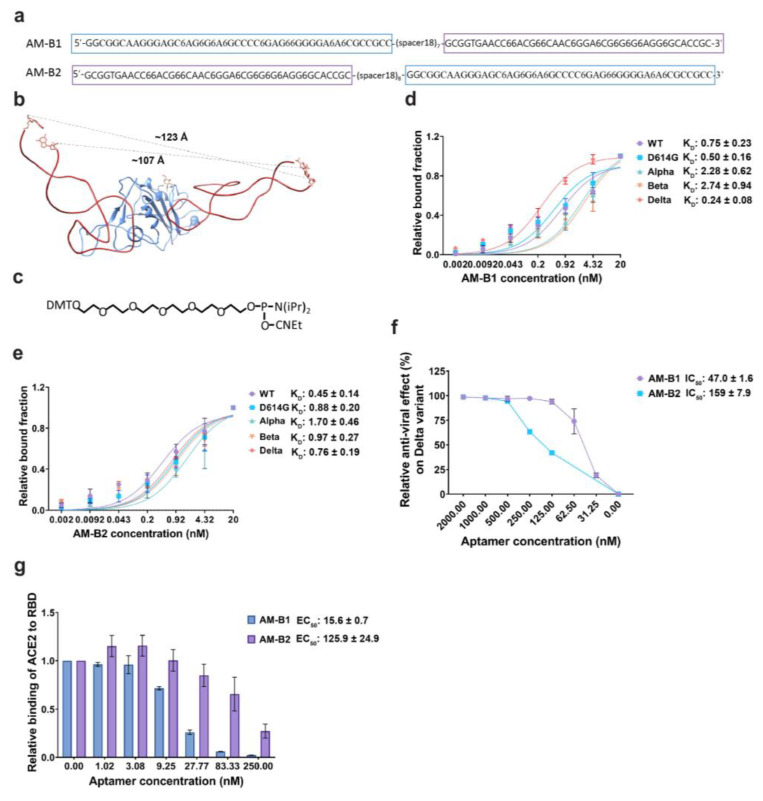
A bivalent aptamer shows strongly augmented antiviral activity. (**a**) Sequences of bivalent aptamers. Aptamers AM032-4 and AM047-6, shown in purple and blue boxes, respectively, are connected together by flexible linkers composed of 7× or 8× spacer 18s (HEGs). (**b**) Distances between the 5′ and 3′ ends of the aptamers AM032-4 and AM047-6 are depicted in the complex structure. Distance information helped in designing bivalent aptamers. (**c**) Chemical structure of phosphoramidite 18 linker that generates spacer18 linker by the polymerization reaction. (**d**,**e**) Determination of binding affinities to the spike proteins of SARS-CoV-2 variants. (**f**) Inhibitory effects of aptamers on the infection of SARS-CoV-2 pseudovirus of the delta variant. (**g**) Inhibitory effects of aptamers on the interaction between ACE2 and RBD. ELISAs were performed to monitor the inhibitory effects. The values presented for K_D_, IC_50_, and EC_50_ were measured in nM.

It is possible to increase the activities of aptamers by connecting two or more aptamers together through a linker(s), which would increase the avidity of aptamers to a target molecule [43,44]. We designed and synthesized two bivalent aptamers, i.e., AM-B1 and AM-B2, using the optimized aptamers AM032-4 and AM047-6. Aptamers AM032-4 and AM047-6 were connected by a long flexible linker [7× or 8× hexaethylene glycol (HEG = Spacer 18)] to obtain bivalent aptamers AM-B1 and AM-B2 based on the three-dimensional structure of the AM032-4–RBD–AM047-6 complex (Figure 6a–c). The 5′ end of AM032-4 was connected with the 3′ end of AM047-6 using a linker of 7× HEG to obtain AM-B1, and the 5′ end of AM047 was connected with the 3′ end of AM032-4 using a linker of 8× HEG to obtain AM-B2 (Figure 6a). AM-B1 and AM-B2 strongly interacted with the spike proteins of several SARS-CoV-2 variants with similar K_D_ values to each other (Figure 6d,e and Supplementary Figure S6a,b). Interestingly, AM-B1 showed stronger inhibitory effects on ACE2 binding (EC_50_ = 15.6 nM) and on pseudovirus infection (IC_50_ = 47.0 nM) than AM-B2 (EC_50_ = 125.9 nM, IC_50_ = 159 nM) (Figure 6f,g). It should be noted that the inhibitory effect of AM-B1 (IC_50_ = 47.0 nM) is higher than those of individual aptamers AM032-4 and AM047-6 (IC_50_ = 230 nM and 139 nM, respectively). The results indicate that a properly arranged bivalent aptamer (e.g., AM-B1) can have stronger inhibitory activity than individual aptamers, but a poorly arranged aptamer (e.g., AM-B2) shows similar inhibitory activity to the stronger monomeric aptamer (e.g., AM047-6).

## 3. Discussion

Herein, we could develop several aptamers having modified nucleotides (Nap-dUs) with high affinities for the spike protein of SARS-CoV-2. Our study focused on the aptamers AM032 and AM047 and their derivatives that bind to the RBD of spike protein. Interestingly, these aptamers showed high affinities (K_D_ = 0.04 nM~10.5 nM) not only to the wild-type spike protein but also to the spike proteins of alpha, beta, and delta variants. This indicates that developing aptamers with a broad spectrum of target molecules is possible by using an appropriate selection process.

To investigate the feasibility of developing aptamers with anti-SARS-CoV-2 activities via a structure-based aptamer-designing approach, we determined the three-dimensional structure of the aptamer/RBD complex using cryo-EM technology. We used RBD instead of a trimeric spike protein due to the flexible nature of RBD relative to the rest of the spike protein [4], which reduces the resolution of the RBD region of the spike in the structural analysis. The three-dimensional structure of the aptamer/RBD complex (resolution up to ~3.43 Å in Supplementary Figure S5f) provided us with the information required for understanding the mechanism of aptamer structure formation and of protein–aptamer interaction. Outstanding features of the aptamer/RBD complex are as follows: (1) The naphthyl group of Nap-dU plays a key role in maintaining the loop structures of both aptamers AM032-0 and AM047-0 that enable specific interactions with RBD (Figure 3 and Figure 4 and Supplementary Table S3). The unusual three-turn structure in aptamer AM032-0 is stabilized by a hydrophobic core composed of seven naphthyl residues, and the L1 and L2 loops in aptamer AM047-0 are maintained by a hydrophobic core composed of four naphthyl residues stretched out from L1 and L2 loops, which provide two naphthyl residues each. (2) The hydrophobic and specific polar interactions between the aptamers and the spike protein are the major forces for the strong and specific associations among the molecules. (3) Base pairing and/or base stacking that resides outside of the loop regions, which participate in aptamer–protein interaction, stabilize the overall structure of the aptamers. These features indicate that the usage of a modified nucleotide (Nap-dU, in this case) can dramatically increase the diversity of shapes of the aptamers by forming hydrophobic cores composed of naphthyl groups. The conformational diversity and the direct participation of the naphthyl group in binding to a target protein seem to increase the possibility of finding aptamers with high affinities, which was experimentally proven previously without knowing the structures [23,24,25,26].

The molecular bases of the antiviral effects of aptamers were revealed by the three-dimensional structure of the aptamer–RBD complex. A large part of the interface of RBD that participates in the interaction with aptamer AM032-0 (F456 and G476~Y505 on RBD) overlaps with that participating in the interaction with ACE2 (G446~Y505 on RBD) [5]. The competition between AM032-0 and ACE2 for the same binding sites on the RBD is the inhibitory mechanism of AM032-0 for the ACE2 binding to the RBD. On the other hand, the inhibitory effect of AM047-0 on the RBD-ACE2 interaction was not attributable to the occupation of the same binding site in RBD by AM047-0 since the aptamer AM047-0 localized to a completely distinct region from the ACE2-binding area (Figure 2c). It is likely that the binding of AM047-0 indirectly inhibits ACE2 binding through the conformational change of RBD.

Our structure-based and optimized aptamers AM032-4 and AM047-6 showed stronger or similar affinities to spike variants compared with the original aptamers (Figure 1 and Figure 5). Moreover, the existence of more stable stem structures due to the incorporation of G-C base pairs at the termini of the optimized aptamers AM032-4 and AM047-6 compared with the original aptamers AM032-0 and AM047-0 was indicated by the higher resolution of the cryo-EM map around the aptamers’ termini of the protein–aptamer complex (Supplementary Figures S3g and S5g). A strong, double-stranded stem structure at the termini of oligonucleotides protects an aptamer from degradation by exonucleases, which are the major nucleases that destroy DNAs or RNAs in the plasma [31,42]. These aptamers can be even further stabilized by replacing the nucleotides forming the stems with locked nucleic acids (LNA) or 2′-O-methyl nucleotides, which are more resistant to nuclease-mediated degradation and form more stable stem structures with a higher melting temperature [45,46,47].

Based on the three-dimensional structure of the RBD–Fab–AM032-4–AM047-6 complex, we designed and synthesized bivalent aptamers containing both AM032-4 and AM047-6 connected by linkers with different lengths. One of the bivalent aptamers (AM-B1) showed an approximately 3-fold higher inhibitory effect than AM047-6 on the infection of pseudovirus SARS-CoV-2 delta. The other bivalent aptamer (AM-B2) showed the same inhibitory effect as AM047-6 on the infection of the delta pseudovirus. These results demonstrate the feasibility of developing highly effective bivalent aptamers based on the structures of aptamers binding to two different regions of a target protein.

While we were carrying out this study, SARS-CoV-2 evolved rapidly to evade host immunity acquired by vaccine treatment and/or by the viral infection of early SARS-CoV-2 variants [12,18,19,20]. In addition, the variants with higher infectivity through their stronger binding affinity to the viral receptor ACE2 outnumbered the earlier variants by their faster-spreading capability [48]. As a consequence, the omicron variants of SARS-CoV-2 have become the most prevalent variants to date [49]. The mutations in SARS-CoV-2 variants are accumulated mostly in the spike protein since it plays a pivotal role in the interaction with ACE2 and is targeted by neutralizing antibodies generated by acquired immunity [50]. The aptamers AM032-0 and AM047-0 and their derivatives have high affinities to the spike proteins of various variants up to the delta variant (Figure 1 and Figure 5), even though these variants contain many mutations in the spike gene that nullify the binding of many neutralizing antibodies [51]. This indicates that these aptamers have a broad spectrum of binding to various spike proteins compared with many monoclonal antibodies. However, unfortunately, these aptamers have reduced binding affinities to the spike of omicron variants that have 15 or more mutations within the RBD region that nullify the binding of many neutralizing antibodies [52] (Supplementary Figure S6e and Supplementary Table S1; AM032-4 and AM047-4 have K_D_ values of 26.7 and 19.9 nM, respectively).

Several mutations exist in the aptamer-binding interfaces in the RBD of omicron variants. For instance, eight (S477N, T478K, E484A, Q493R, G496S, Q498R, N501Y, and Y505H) [53] and seven (S477N, T478K, E484A, F486V, Q498R, N501Y, and Y505H) [54] mutations exist in the AM032-binding interfaces of omicron B.1.1.529 and BA.4/5, respectively (Supplementary Figure S6c and Table S4). A slight movement (1.5 to 2.4 Å) of aromatic amino acids in the loop of omicron variants composing the AM032-binding interface and several mutations in amino acids that participate in aptamer–RBD interaction seem to attribute to the reduced affinity of the omicron spike to AM032 (Supplementary Figure S6c). Moreover, we confirmed the importance of E484, F486, and Q493 residues in aptamer binding by testing the effect of mutations in RBD (Figure 3d). Three (S371L, S373P, and S375F) and four (S371L, S373P, S375F, and T376A) mutations exist in the AM047-binding interfaces of omicron B.1.1.529 and BA.4/5, respectively (Supplementary Table S2). The presence of these mutations causes conformational differences in the AM047-binding interface of omicron, as shown in Supplementary Figure S6d, thereby explaining the reduced binding of omicron to AM047. Moreover, we confirmed the importance of residues 373 and 375 of the spike in the interaction with AM047 by reverting these mutations to the wild type (P373S and F375S) and testing the affinity of the reverted RBD for AM047. The reversion mutations of omicron BA.4/5 restored the binding affinity to AM047-0 almost completely (Figure 4d).

## 4. Materials and Methods

### 4.1. Cloning, Expression, and Purification of Proteins

In order to synthesize the spike proteins of SARS-CoV-2, we used a baculovirus gene expression system [35,36]. The DNA fragment encoding the ectodomain of the spike protein (designated as WT-S, residues 1–1208) of SARS-CoV-2 containing two proline substitutions at 986–987 amino acid positions, a furin cleavage site mutation (RRAR to GSAS at position of 682–685 residues), and a C-terminal trimerization motif [4] was amplified by PCR using a plasmid kindly provided by Prof. Jason S. McLellan of University of Texas at Austin as a template. A TEV protease cleavage site was newly included during PCR using a proper primer. The synthesized DNA fragment was cloned into a p42 vector (a modified pFastBac) containing 6xHis and a Flag-tag [35,36]. To create a plasmid encoding D614G mutation in the spike gene, site-directed mutagenesis (SDM) was performed. Other DNA fragments encoding the spike genes of alpha, beta, delta, and omicron variants with two proline substitutions and the furin cleavage site mutations were synthesized by Gene Universal and cloned into the p42 vector, as described for WT-S construction. In order to produce plasmids encoding human ACE2 protein (residues 19-615) and the polypeptide corresponding to the RBD of the spike (319−541 aa) with an N-terminal honeybee melittin (HBM) signal sequence [55], C-terminal 6xHis-tag, and an Fc-tag, DNA fragments corresponding to the genes were amplified by PCR using the plasmids kindly provided by Prof. Jason S. McLellan as templates. The synthesized DNA fragments were cloned into the p42 vector as described above.

Recombinant proteins were produced using a baculovirus-expression system as previously described [35,36]. Sf9 insect cells (2 × 10^6^ cells/mL) were inoculated with baculoviruses expressing recombinant proteins and cultivated for three days, and then, culture media were collected. The culture media were centrifuged at 6000 rpm for 10 min to remove cell debris and then filtered through a 0.45 µm syringe filter (Sartorius). The filtrate was passed through a His-tag purification resin (Roche) for protein binding and then washed with wash buffer (50 mM Tris-HCl (pH 8.0), 200 mM NaCl, and 20 mM imidazole), and then, the proteins were eluted in elution buffer (50 mM Tris-HCl (pH 8.0), 200 mM NaCl, and 200–300 mM imidazole). Eluted fractions were pooled, concentrated, and injected into a Superose^®^ 6 Increase 10/300 GL column (GE Healthcare) for size-exclusion chromatography (SEC) in a buffer of 20 mM Tris-HCl (pH 8.0) and 200 mM NaCl. Finally, SEC fractions were pooled, concentrated, and stored at −70 °C for further experiments. RBD protein was purified (Supplementary Figure S1b,f) by the same method with a final buffer of 25 mM Tris-HCl (pH 7.5) and 150 mM NaCl in SEC (Superdex 200 increase 10/300 GL, GE healthcare). Other RBD mutants of the wild-type (Y369A, F456A, F486A, and Y489A), delta (E484A, Q493R, and E484A + Q493R), and omicron BA.4/5 (P373S and P373S + F375S) varieties were generated by SDM and purified by the same method as wild-type RBD. Fc-tagged ACE2 proteins were purified (Supplementary Figure S1c,g) by passing the filtrate through protein A resin, washed with wash buffer (50 mM Tris-HCl (pH 8.0) and 200 mM NaCl), and eluted with buffer (100 mM glycine (pH 3.0) and 150 mM NaCl). The pH of the eluent was raised to ~pH 8.0 by adding 1M Tris-HCl (pH 9.0), and then, the buffer was exchanged for 50 mM Tris-HCl (pH 8.0) and 200 mM NaCl. Buffer-exchanged protein was subsequently purified by SEC (Supplementary Figure S1g). 

For the expression and purification of Fab (PDB ID: 6XDG) [38], genes for the heavy and light chains of Fab were synthesized by Twist Bioscience. A signal sequence was added at the N-terminal ends of both chains, and an alpha-tag [56] with a thrombin cleavage site and an 8x His-tag were added at the C-terminal ends of the heavy and light chains, respectively. Genes were cloned into the pAcGP67 vector for the generation of recombinant baculovirus [57]. Hi5 insect cells were co-infected with baculoviruses expressing heavy and light chains. Upon clarification of cells by centrifugation, the secreted Fab complex was purified by Ni-affinity column (Roche) with wash buffer (20 mM Tris-HCl (pH 8.0), 200 mM NaCl, and 1 mM PMSF) and gradient elution buffer (20 mM Tris-HCl (pH 8.0), 200 mM NaCl, and 100-500 mM imidazole). Elution fractions were pooled and purified by SEC (Superdex 200), and peak fractions were concentrated and stored at −80 °C. 

### 4.2. Preparation of Surrogate Virus

Surrogate baculovirus preparation was performed as described previously [36]. Briefly, the ectodomain (polypeptide spanning the outer part of the viral envelope: 1-1208 residues) of the SARS-CoV-2 spike conjugated with the foldon domain of the T4 phage [4] was cloned in front of the G-stem of the vesicular stomatitis virus (VSV) with a C-terminal Flag-tag as a single open-reading frame in a p42 vector. The VSV G-stem was used as an anchor on the viral envelope to display the spike protein on the surface of the baculovirus as described previously [58]. The surrogate baculovirus was generated using a baculovirus-expression system. At three days after infection, the surrogate viruses in Sf9 culture media were collected by centrifugation (500× *g* for 5 min) and cleared by a 0.2 μm syringe filter. The viruses in the media were precipitated by ultra-centrifugation (100,000× *g* for 2 h) in a 20% sucrose gradient in a sucrose gradient buffer (5 mM Tris-HCl (pH 8.0), 100 mM NaCl, and 1 mM EDTA). The surrogate virus pellet was resuspended in cold PBS buffer (137 mM NaCl, 2.7 mM KCl, 10 mM Na_2_HPO_4_, and 1.8 mM KH_2_PO_4_) with 2.5% glycerol and stored at −70 °C.

### 4.3. Systematic Evolution of Ligands by Exponential Enrichment (SELEX)

The SELEX procedures were performed as described by Kwon et al. and Narayan et al., with a few modifications [35,36]. The SELEX process is schematically illustrated in Supplementary Figure S2a, depicting positive and negative selection processes. Protein-based SELEX (left) and virus-based SELEX (right) were executed alternatively. For the enrichment of aptamers specifically interacting with the spike protein of SARS-CoV-2, positive and negative selection processes were included. For positive selection, monomeric spike protein mS (Sino Biological Inc., Wayne, PA, USA), purified spike protein of SARS-CoV-2 (WT-S), and a surrogate virus containing the spike of SARS-CoV-2 (SARS-CoV-2 surrogate virus) were used in the viro-SELEX. For negative selection, a mixture of spike proteins (purchased from Sino Biological Inc.) of human betacoronaviruses (HCoV HKU1 and HCoV OC43) were used in the SELEX (Supplementary Figure S2a). Among the 12 rounds of SELEX, the seventh round was performed with a SARS-CoV-2 surrogate virus (in positive selection) and baculovirus (in negative selection). The ssDNA library contains 90-mer DNAs composed of a randomized nucleic acid sequence (40-mer) in the center flanked by 25-mer constant regions at both ends. A modified nucleotide, i.e., 5-[N-(1-naphthylmethyl) carboxamide]-2′-deoxy uridine (Nap-dU), was used instead of natural nucleotide deoxythymidine for the random sequence of the library. The sequence pattern of library DNAs is similar to 5′-ATATATATCGAGCGTCCTGCCTTTG-40N-CACCGACAGCCACCCAGAAAAAAAA-3′, where 40N represents 40 random sequences including a modified nucleotide (Nap-dU).

To perform aptamer refolding, an aptamer library mixture (1 nmole) dissolved in S buffer (40 mM HEPES (pH 7.5), 102 mM NaCl, 5 mM KCl, 5 mM MgCl_2_, and 0.05% Tween-20) was heated at 95 °C for 5 min, followed by slow cooling to 37 °C at a rate of 0.1 °C/s. To remove unwanted magnetic beads and His-tag binders, aptamer library DNAs were pre-incubated with 6xHis-tag-associated Talon beads (Invitrogen, Carlsbad, CA, USA). The bead-bound DNAs were separated by applying a magnetic field, and the remaining solution with unbound DNAs was incubated with purified proteins (50 pmol) for 60 min at 37 °C. Next, the solution was incubated with Talon beads for 10 min with agitation, and the beads were collected using a magnetic field and washed three times with S buffer. The target-protein-bound aptamers were eluted from the beads using a 2 mM NaOH solution. The eluted DNAs were then amplified by PCR using a 5′-primer (5′-ATATATATCGAGCGTCCTGCCTTTG-3′) and a biotin-conjugated 3′-primer [5′-(2x) Biotin-TTTTTTTTCTGGGTGGCTGTCGGTG-3′]. After the PCR, biotin-conjugated DNAs were trapped on Myone SA beads (Invitrogen), and 20 mM NaOH elution buffer was used to elute the positive-sense DNAs. In order to synthesize positive-sense aptamers containing Nap-dU, the negative-sense DNAs trapped on the beads were used as templates, and the beads were incubated in a polymerization reaction buffer comprising DNA polymerase, dATP, dGTP, dCTP, and Nap-dUTP. The synthesized aptamers were used for the next round of SELEX. The SELEX process was executed in the absence or presence of dextran sulfate (DxSO_4_). A decreasing amount of spike protein (down to 0.005 pmol) and an increasing amount of DxSO_4_ (up to 10 μM) were added in the binding step and/or after the binding of protein with a DNA pool. The ssDNAs, enriched in the 11th round of SELEX, were amplified by PCR using the 5′- and 3′-primers and purified using a PCR purification kit (Qiagen, Germany). The purified DNAs were cloned and sequenced (Solgent, South Korea). Alternatively, the amplified DNAs were sequenced using a next-generation sequencing (NGS) method.

### 4.4. SARS-CoV-2 Spike Aptamer Synthesis

The aptamers were synthesized by Aptamer Sciences Inc. as described previously [35,36].

### 4.5. Measurement of the K_D_ Values of Aptamers

A filter binding method as described by Gold et al. (2010) was used to determine the equilibrium dissociation constant (K_D_) between an aptamer and a protein [26]. In short, [^32^P] radioisotope and [^32^P]-ATP (Perkinelmer, Waltham, MA, USA) were used to label the 5′ ends of aptamers using T4 polynucleotide kinase (Takara Bio Inc.). The radiolabeled aptamers were diluted to 20,000 cpm in 200 μL of S buffer and then heated at 95 °C for 3 min and cooled slowly to 37 °C at a rate of 0.1 °C/s. The radiolabeled aptamers were incubated with various amounts of the target proteins (20 nM to 2.1 pM with a 4.6-fold dilution) at 37 °C for 30 min. Then, 1/30 of them were spotted on a non-charged nylon membrane. Zorbax resin (Agilent, Santa Clara, CA, USA) in H_2_O (5.5 μL, 400 mg/mL) was added to the aptamer–protein solution and mixed with a thermomixer for 1 min at 1300 rpm. The target protein and radiolabeled aptamer mixer were then applied to a polyvinylidene difluoride (PVDF) filter plate and washed, and the radioactivity remaining on the filter plate and nylon membrane was measured with a phosphor-imager (Amersham Typhoon 5 Biomolecular Imager, GE healthcare, Japan). The radioactivity in each slot was normalized to that of the negative control ([^32^P]-labeled library DNAs) slot. The K_D_ values of aptamers were calculated by plotting the data in one site-specific binding model using GraphPad Prism software, version 9.5. 

### 4.6. Protein-Binding Competition Assay

Competition between aptamers for binding to SARS-CoV-2 spike proteins was monitored as described by Kwon et al. [35]. [^32^P]-labeled (hot) aptamers (2000 cpm) were incubated with SARS-CoV-2 spike protein (mS) in the presence of unlabeled (cold) aptamers (25 pmole) as competitors at 37 °C for 30 min, and then, aptamer–protein complexes were captured by Zorbax resin. The amount of radio-labeled aptamers on the Zorbax resin was measured with a phosphor-imager (Amersham Typhoon 5 Biomolecular Imager).

### 4.7. Enzyme Linked Immunosorbent Assay (ELISA)

The aptamer activity in blocking the interaction between RBD and ACE2 was measured by competitive ELISA. In short, 100 μL of RBD protein (1 μg/mL) was pre-coated on Maxi binding ELISA plates (SPL Life Sciences) by incubating overnight at 4 °C. The next day, the plate was blocked with 5% BSA in PBST buffer (PBS buffer with 0.05% Tween-20) for 1 h at 37 °C and then washed three times with PBST buffer (200 μL/well). Aptamers were heated at 95 °C for 5 min and slowly cooled to 37 °C at a rate of 0.1 °C/s. Aptamers were serially diluted in PBST with 2 mM MgCl_2_ (50 μL) and mixed with 50 ng of ACE2 (50 μL). This reaction mixture (100 μL) was applied to each well of the ELISA plate and incubated for 15 min at 37 °C. The wells were washed three times with PBST buffer, and then, HRP-conjugated anti-human IgG1-HRP antibody (Sigma-Aldrich, St. Louis, MO, USA) at a dilution of 1:40,000 was added to the well and incubated at 37 °C for 1 h. After washing, the color-development reaction was carried out by adding 100 μL of 3,3′,5,5′-tetramethylbenzidine (TMB) substrates (Sigma-Aldrich, T0440), and the reaction was stopped by adding 100 μL of 1N H_2_SO_4_. Optical density at 450 nm (OD_450nm_) was read in a microplate reader (Tecan, Infinite M200pro, Männedorf, Switzerland). For all the experiments, triplicate wells were used.

### 4.8. Measurement of Antiviral Activities of Aptamers

Pseudoviruses based on an HIV backbone containing SARS-CoV-2 spike proteins of D614G and delta variants with a deletion (21 aa) at the C-termini were used to measure the antiviral effects of aptamers [31,37]. Briefly, for the production of pseudoviral particles, HEK293T cells were transfected with five plasmids: (1) pHAGE-CMV-Luc2-IRES-ZsGreen-W (NR-52516, BEI resources), (2) pHDM-Hgpm2 (NR-52517, BEI resources), (3) pHDM-tat1b (NR-52518, BEI resources), (4) pRC-CMV-Rev1b (NR-52519, BEI resources), and (5) pHDM-SARS-CoV-2 D614G (NR-53765, BEI resources) or a derivative of NR-53765 containing the SARS-CoV-2 delta variant gene. At 18–24 h after transfection, the media were changed, and the cells were further cultivated for 40 h. The media were harvested, filtered through a 0.45 μm pore-sized filter, and stored at −70 °C as a pseudovirus stock. For antiviral activity assays, the media containing pseudoviral particles were incubated with different amounts of aptamers (1000 nM, 500 nM, 250 nM, 125 nM, 62.5 nM, 31.2 nM, and 0 nM) at 37 °C for 60 min and then applied to 293T-ACE2 cells that were cultivated for 12 h after seeding of ~2 × 10^4^ cells/well on a 96-well plate. After 6 h of incubation, the media were changed with fresh DMEM media with 10% FBS, and the cells were further cultivated for 48 h. The media were discarded, and 30 μL of passive lysis buffer (Promega, Madison, WI, USA) was applied to each well, and the plate was incubated for 5 min. Firefly luciferase substrate (E1501, Promega, Madison, WI, USA) was added to each well, and the luciferase activity in each well was measured for 1 s using a Tecan Infinite M200 Pro reader.

### 4.9. Cryo-EM Grid Preparation

RBD and Fab proteins were mixed at a 1:1.3 molar ratio, incubated on ice for one hour, and then fractionated by size-exclusion chromatography (Supplementary Figure S3a). Two aptamers (1.2-fold molar excess of each aptamer to RBD) were added to the RBD–Fab complex protein (1 mg/mL). The mixture was supplemented with 0.15% amphipol A8-35 (Anatrace, Maumee, OH, USA) and 1 mM MgCl_2_ and incubated for 1 h on ice. Detergent Cymal-6 (Anatrace) of 0.003% was added to the sample before the vitrification. The protein–aptamer complex (2.5 μL) was applied to freshly prepared glow-discharged Quantifoil R1.2/1.3 300 or 400 mesh copper grids (EMS, Hatfield, PA, USA). The grids were blotted for 5 s at 4 °C with 100% humidity and plunge-frozen in liquid ethane using Vitrobot Mark IV (FEI). Cryo-EM data were collected using a 300 kV Titan Krios Electron Microscope equipped with a Gatan K3 BioQuantum direct electron detector in a fast mode with a magnification of 105,000×. For the complex of RBD–Fab–AM032-0–AM047-0, data were collected with an accumulated dose of 50 electrons/Å^2^ and defocus range of −0.8 to −2.0 µm. In the case of the RBD–Fab–AM032-4–AM047-6 complex, data were collected with an accumulated dose of 60 electrons/Å^2^ and defocus range of −0.7 to −1.6 µm.

### 4.10. Data Processing for RBD–Fab–AM032-0–AM047-0 Complex

The data processing workflow for the RBD–Fab–AM032-0–AM047-0 aptamer complex is summarized in Supplementary Figure S3. Briefly, 11,743 movies (dataset 1) of dose-fractionated image stacks were imported into cryoSPARC v3.3.2 [39], and then patch motion correction was used to align and dose weight the images. CTF parameters were calculated by means of patch CTF estimation. Micrographs with low quality were removed. The remaining 10,880 micrographs were used for data processing. The protein–aptamer particles were picked and extracted using the Topaz method [59]. An additional dataset was collected to increase the number of particles. Full-frame motion correction was used for aligning and dose weighting the second dataset, and then, patch CTF estimation was used to calculate the CTF parameters. The remaining 8720 micrographs were used for data processing after eliminating the low-quality micrographs. Particles from the two data sets were combined and underwent an additional round of 2D classification, which selected 1,837,985 particles. The particles were re-extracted without binning, duplicate particles were removed, and they were once again subjected to 2D classification. In total, 1,085,102 particles were selected from this round of 2D classes and used for two rounds of heterogeneous refinement. After heterogeneous refinement, 387,167 particles were subjected to non-uniform refinement, which yielded a map with a global resolution of 3.82 Å. Global CTF refinement and non-uniform refinement resolution improved the map to 3.8 Å. The non-uniform refined model was reconstructed in 3DFlex refinement [60] in cryoSPARC v4.1.0 [39], and the final resolution was improved to 3.32 Å for the overall map. 

### 4.11. Data Processing for RBD–Fab–AM032-4–AM047-6 Complex

The data processing workflow for the RBD–Fab–AM032-4–AM047-6 aptamer complex is summarized in Supplementary Figure S5. CryoSPARC v4.1.0 [39] was used to import the dose-fractionated image stacks from 10,018 movies. Images were aligned and dose-weighted using patch motion correction, and CTF parameters were calculated using patch CTF estimation. Micrographs with low quality were removed. The remaining 9480 micrographs were used for data processing. The protein–aptamer particles were picked and extracted using the Topaz method. Selected particles from 2D classes were subjected to heterogeneous refinement. After heterogeneous refinement, 230,681 particles showing the complex were refined using non-uniform (NU) refinement. Particles from the NU refinement were further corrected for motion using local motion correction, global CTF refinement, and local CTF refinement, and then, 212,355 particles were refined again using non-uniform refinement that resulted in a 3.29 Å resolution map. The map from the NU refinement was next subjected to 3DFlex reconstruction [60], which improved the map to 3.43 Å resolution. 

### 4.12. Model Building and Refinement

The auto-DRRAFTER program [61] was used to obtain the initial models of the aptamers using conventional nucleotides (see Supplementary Notes for detail). Some of the dU nucleotides in the initial model were replaced with Nap-dU using Coot [41]. Initial structures for the RBD and Fab parts were built using previously reported structures obtained from PDB IDs 7KGJ [40] and 6XDG [38], respectively, and were fitted to the cryo-EM density map using the Chimera [62] program. The fitted models of RBD, Fab, and aptamers were combined and adjusted again with the cryo-EM density map in Chimera [62]. Next, multiple rounds of real space refinement and manual modeling building were performed using Phenix [63] and Coot [41] programs to prepare the final model (Supplementary Figure S3h). The details of the refinement properties of each complex are also presented in Supplementary Table S2. The figures related to structure were built in Chimera [62].

### 4.13. Accession Numbers

The cryo-EM density maps and models of the RBD–Fab–AM032-0–AM047-0 complex (EMDB: EMD-35930, PDB code: 8J1Q) and of the RBD–Fab–AM032-4–AM047-6 complex (EMDB: EMD-35945, PDB code: 8J26) have been deposited to the EMDB and PDB. 

## 5. Conclusions

This study clearly demonstrates that the acquisition of aptamers with high affinities to the viral envelope protein, i.e., the spike protein, is possible using a modified nucleotide and a carefully designed SELEX process. We revealed the molecular bases of aptamers’ activities (protein binding and inhibition of virus infection) through the cryo-EM structure of the aptamer/RBD complex, and we also showed the possibility of aptamer optimization using a structure-based aptamer-designing approach. It may also be possible to improve the aptamers to have higher affinities to a target molecule by modifying the aptamers based on the structure of the aptamer–target complex in the future. Finally, we demonstrated that the development of bivalent aptamers, which have higher activity than individual aptamers, is possible by analyzing the structure of the aptamer–target complex.

## Data Availability

Data will be provided upon request.

## Supplementary Materials

The following supporting information can be downloaded at: https://www.mdpi.com/article/10.3390/molecules28124645/s1, Figure S1: Protein expression and purification, Figure S2: Schematic diagram of SELEX process and characterization of aptamers, Figure S3: Workflow of data processing for determining the structure of RBD–Fab–AM032-0–AM047-0 complex, Figure S4: Optimization of aptamers AM032-0 and AM047-0, Figure S5: Workflow of data processing for determining the structure of RBD–Fab–AM032-4–AM047-6 complex, Figure S6: Characterization of bivalent aptamers AM-B1 and AM-B2; Table S1: Binding affinities of the aptamers, Table S2: Summary of the cryo-EM data collection and refinement, Table S3: Nucleotides in aptamers and amino acids residues at the interacting interfaces with a distance cut-off of 4 Å, Table S4: Mutations in RBD observed at the binding surface of aptamers AM032-0 and AM047-0. The reference [64,65,66,67,68,69,70] is in the Supplementary Materials.

## Sample Availability

Not available.
