# Supplementary material for: Structure-Guided Development of Bivalent Aptamers Blocking SARS-CoV-2 Infection
Source: Molecules. 2023 Jun 8;28(12):4645. doi: 10.3390/molecules28124645 (PMC10303109; doi:10.3390/molecules28124645)
Supplement: Supplementary file 1 [file molecules-28-04645-s001.zip › molecules-2436899-supplementary.pdf]

Supplementary Information

Article

Structure-Guided Development of Bivalent Aptamers Blocking SARS-CoV-2 Infection

Md Shafiqur Rahman [1], Min Jung Han[1], Sang Won Kim[1], Seong Mu Kang[1], Bo Ri Kim[1], Heesun Kim[2], Chang Jun Lee[1], Jung Eun Noh[1], Hanseong Kim[1], Jie-Oh Lee[1,*] and Sung Key Jang[1,2,*]

1 Department of Life Sciences, POSTECH Biotech Center, Pohang University of Science and Technology, 77 Cheongam-ro, Nam-gu, Pohang-si, Gyeongsangbuk-do, Rep. of KOREA 37673.

2 Division of Integrative Bioscience & Biotechnology, POSTECH Biotech Center, Pohang University of Science and Technology, Nam-gu, Pohang-si, Gyeongsangbuk-do, Rep. of KOREA 37673.

* Correspondence: sungkey@postech.ac.kr; jieoh@postech.ac.kr.com

Supplementary Figures

Supplementary Figure S1

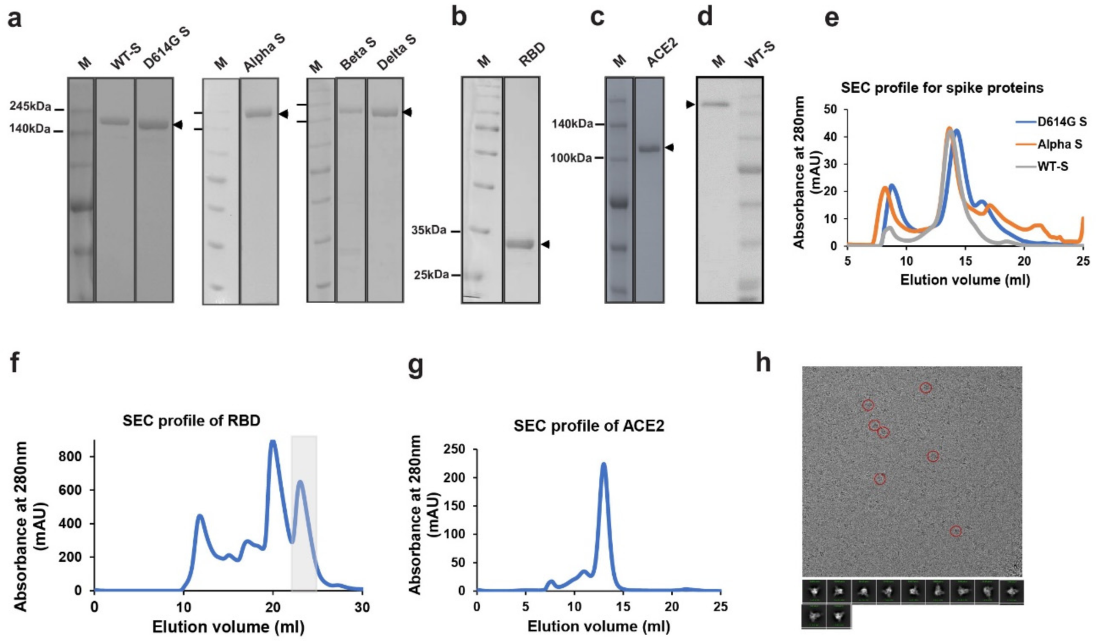

Figure S1. Protein expression and purification. (**a-c**) The SDS-PAGE shows purified spike proteins (ectodomain) of wild type (WT-S), D614G (D614G S), alpha (Alpha S), beta (Beta S), and delta variants (Delta S) (a), RBD of WT-S (b), and ACE2 (c). The purified protein bands are indicated by arrowheads. (**d**) The existence of SARS CoV-2 spike proteins in surrogate baculoviruses was confirmed by Western blotting using an anti-Flag antibody (Sigma-Aldrich). (**e**) Representative size exclusion chromatographic (SEC) profiles of spike proteins of D614G S, Alpha S, and WT-S using Superpose 6, 10/300 GL column (GE Healthcare). (**f**) Representative SEC profiles of RBD using Superdex 200 increase, 10/300 column (GE Healthcare). The peak of RBD is depicted by a gray box. (**g**) SEC profile of ACE2. (**h**) A representative electron micrograph (top) and 2D class average (bottom) of D614G S. Trimeric particles are depicted by red circles in the cryo-EM image.

Supplementary Figure S2

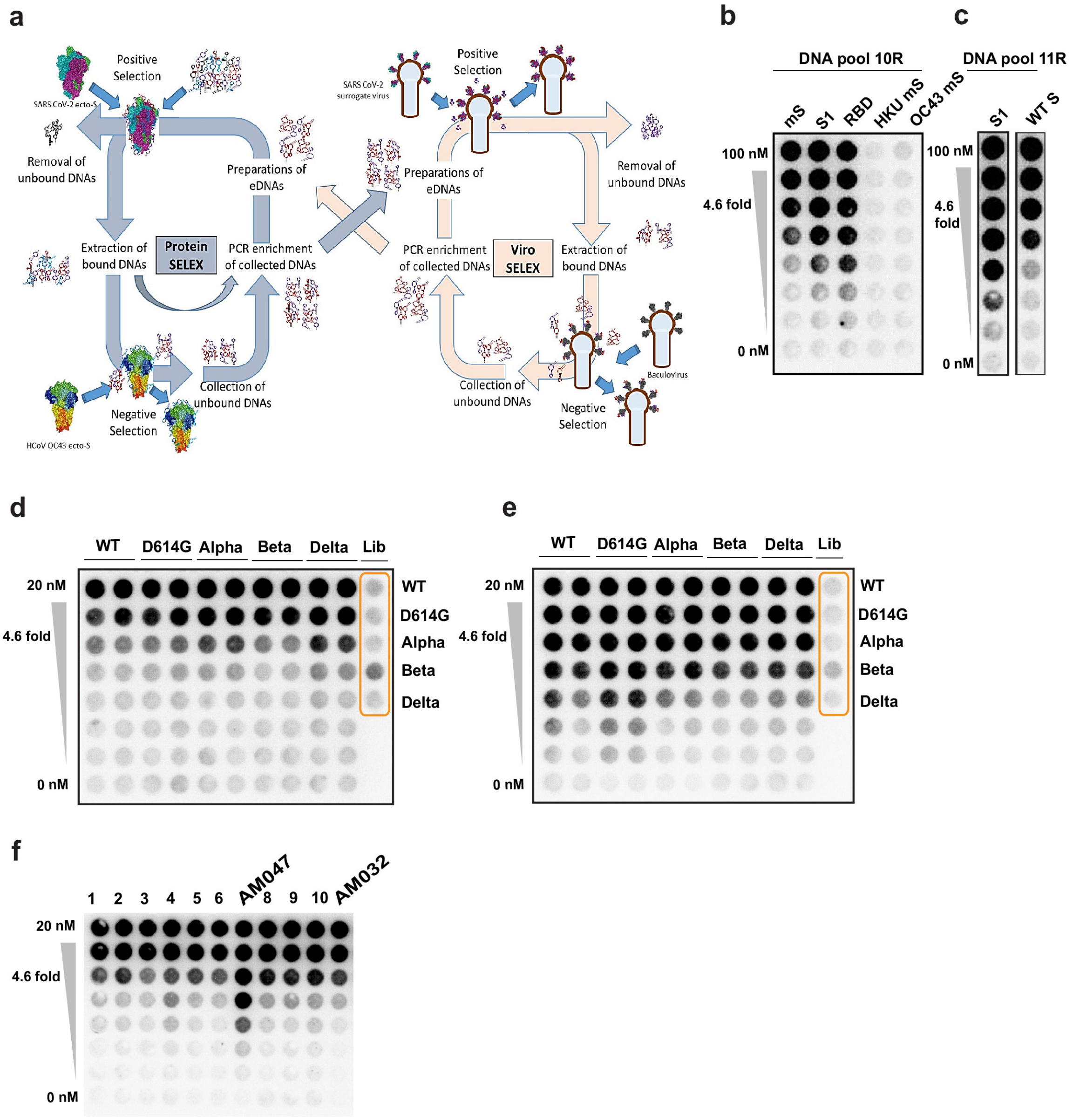

Aptamers	1	2	3	4	5	6	AM047	8	9	10	AM032
K_D	2.37	2.12	2.30	2.80	2.57	2.21	0.16	2.23	2.45	1.95	2.83

Figure S2. Schematic diagram of SELEX process and characterizations of aptamers. (**a**) Schematic diagram of SELEX. Positive and negative selection processes were executed by both protein-based SELEX (protein-SELEX, left) and virus-based SELEX (viro-SELEX, right). The structures of trimeric SARS-CoV-2 spike (PDB ID: 6VSB) and HCoV OC43 spike (PDB ID: 6OHW) are depicted in this diagram. The pictures are not drawn to scale. (**b**) Filter binding assays using the DNA pool of the 10th round in SELEX. Monomeric spike containing both S1 and S2 domains

(mS), S1 domain (S1), and RBD domain (RBD) of wild type spike protein were used in the filter binding assays. The mS proteins of human betacoronaviruses (HKU1 and OC43) were used as negative controls. (**c**) Filter binding assays using the DNA pool of the 11th round in SELEX. S1 domain (S1) and WT-S were used in the filter binding assays. (**d, e**) Filter binding assays of aptamers AM032-0 (**d**) and AM047-0 (**e**) with spike proteins of SARS-CoV-2 variants. Experiments were performed in duplicates. Lib: aptamer library. (**f**) Filter binding assays of different aptamers against wild-type spike protein. The K_D values are depicted in nanomolar (nM) below the filter binding assay.

Supplementary Figure S3

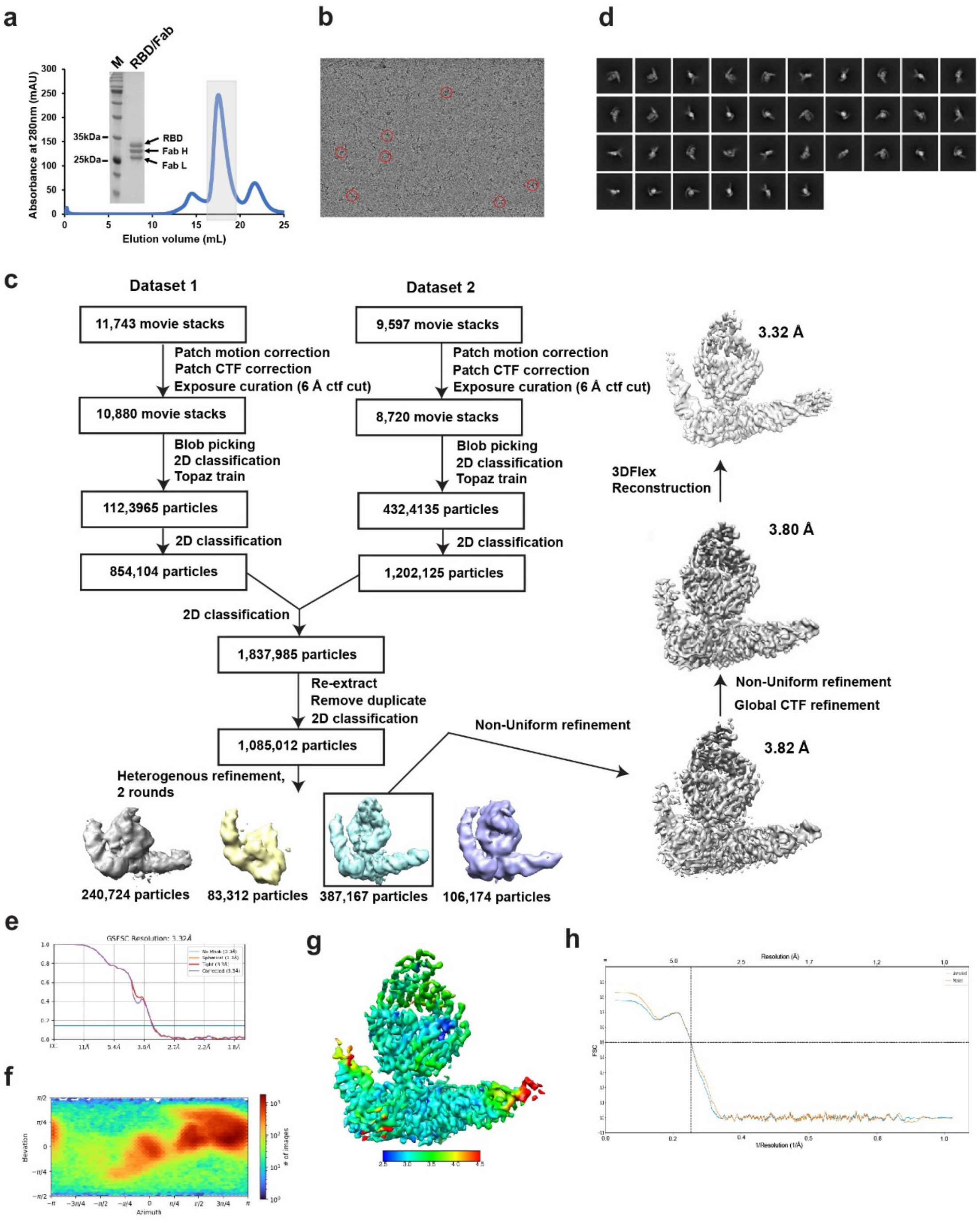

Figure S3. Workflow of data processing for determining the structure of RBD-Fab-AM032-0-AM047-0 complex. (**a**) SEC profile of RBD/Fab complex. The peak fraction in the gray box was collected and used for cryo-EM experiments. The proteins in the peak were analyzed by SDS-PAGE. (**b**) A representative micrographic image of the RBD-Fab-AM032-0-AM047-0 complex.

Red circles indicate complex particles. (**c**) Workflow of data processing. (**d**) Representative 2D class averages of RBD-Fab-AM032-0-AM047-0 complex. (**e**) Gold standard Fourier shell correlation (FSC) curves for the finally refined map at 3.32 Å. (**f**) Angular distribution of protein particles. (**g**) Local resolution of the cryo-EM map (front view). (**h**) Map-to-model FSC curves between the refined structure and the map.

Supplementary Figure S4

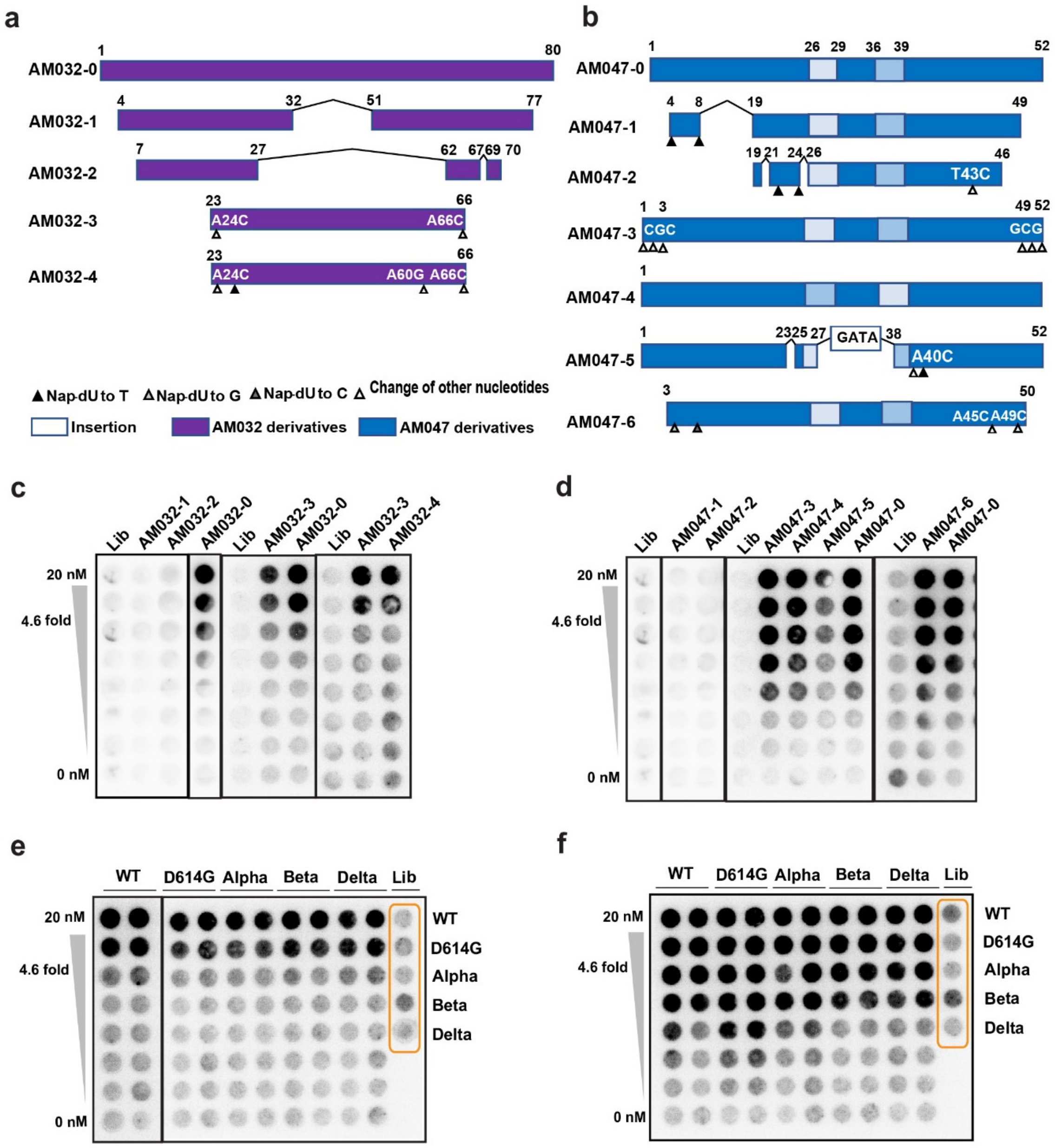

Figure S4. Optimization of aptamers AM032 and AM047. (**a, b**) Schematic diagrams of aptamers AM032 (a) and AM047 (b) derivatives are shown. The pictures are not drawn to scale. (**c, d**) Filter binding assays of aptamers AM032 (**c**) and AM047 (**d**) derivatives were performed with wild-type RBD. (**e, f**) Filter binding assays of aptamers AM032-4 (e) and AM047-6 (f) with spike proteins of SARS-CoV-2 variants. Aptamer library DNAs (orange box) were incubated with spike proteins of SARS-CoV-2 variants (20 nM) as a control. Lib: aptamer library.

Supplementary Figure S5

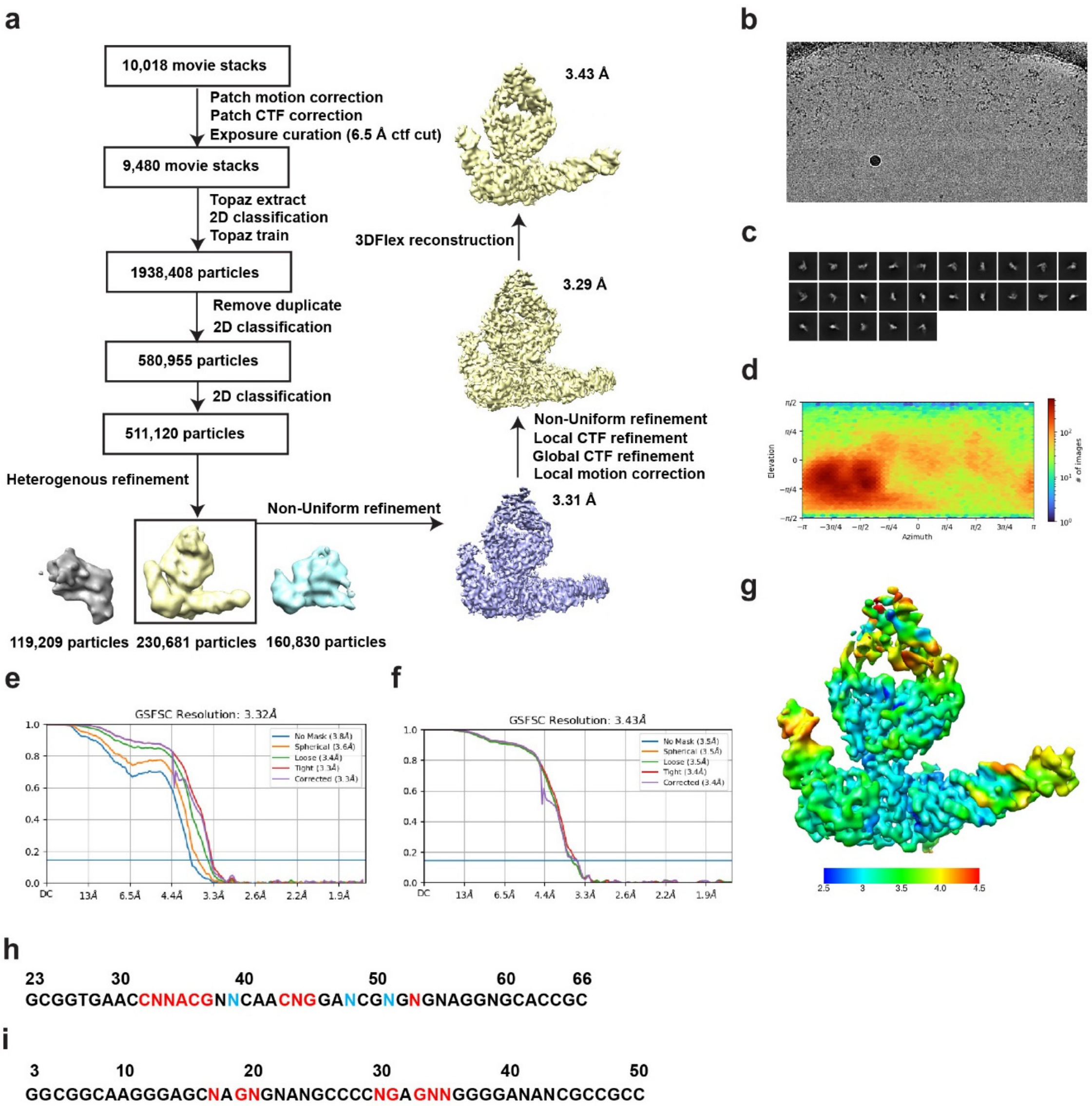

Figure S5. Workflow of data processing for determining the structure of RBD-Fab-AM032-4-AM047-6 complex. (**a**) Workflow of data processing for RBD-Fab-AM032-4-AM047-6 complex. (**b**) A representative micrographic image of RBD-Fab-AM032-4-AM047-6 complex. (**c**) Representative 2D class averages of RBD-Fab-AM032-4-AM047-6 complex. (**d**) Angular distribution of protein particles. (**e, f**) FSC curves for refined maps resulting in 3.32 Å resolution after non-uniform refinement (**e**) and 3D flexible reconstruction resulting in 3.43 Å resolution (**f**). (**g**) Local resolution of the cryo-EM map (front view). (**h, i**) Sequences of the aptamers AM032-4 (**h**) and AM047-6 (**i**). Structurally and functionally important residues are depicted in blue and red colors, respectively. N: Nap-dU = 5-[N-(1-naphthylmethyl) carboxamide]-2'-deoxyuridine, A: 2'-deoxyadenosine, T: 2'-deoxythymidine, G: 2'-deoxyguanosine, C: 2'-deoxycytidine.

Supplementary Figure S6

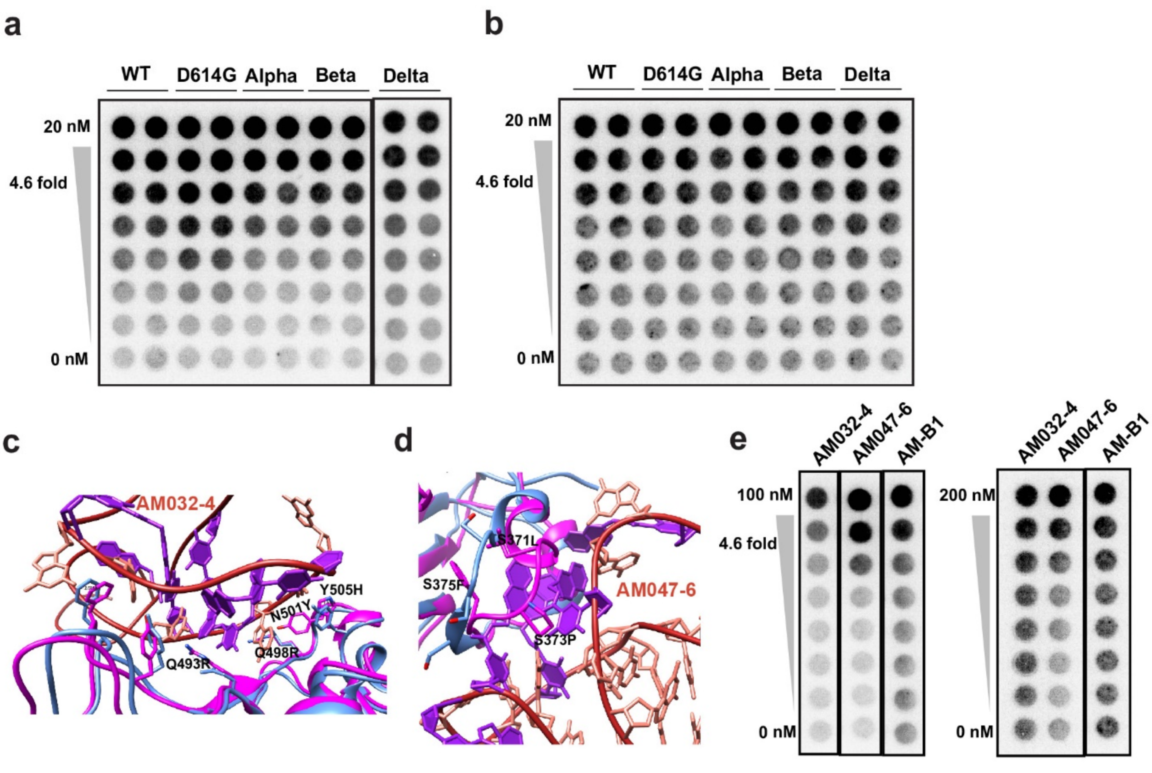

Figure S6. Characterizations of bivalent aptamers AM-B1 and AM-B2. (**a, b**) Filter binding assays of AM-B1 (a) and AM-B2 (b) with the spike proteins of SARS-CoV-2 variants. (**c, d**) Superimposition of RBD-aptamer complex with omicron RBD (PDB ID: 7YAD, Cyan). The binding sites of AM032-4 (**c**) and AM047-6 (**d**) are shown. The residues having prominent differences between wild-type and omicron RBDs are shown in stick model and labeled. The RBD of RBD-aptamer complex and the omicron RBD are depicted in light blue and magenta colors,

respectively. (**e**) Filter binding assays of aptamers with the RBD of omicron BA.4/5 RBD (left) and the spike protein of omicron B.1.1.529 (right).

Supplementary Tables

Table S1. Binding affinities of the aptamers

Protein	Binding Affinity (K_D) in nanomolar (nM)				
	**AM032-0**	**AM032-4**	**AM047-0**	**AM047-6**	**AM-B1**
mS	5.98	ND	2.27	ND	ND
S1	7.62	ND	0.42	ND	ND
RBD	0.78	ND	0.02	ND	ND
WT S	9.51 ± 1.43	8.15 ± 1.27	0.11 ± 0.02	0.11 ± 0.02	0.75 ± 0.23
D614G S	5.07 ± 0.40	8.71 ± 1.79	0.04 ± 0.01	0.04 ± 0.01	0.50 ± 0.16
Alpha S	4.76 ± 0.37	6.47 ± 1.35	0.17 ± 0.04	0.11 ± 0.04	2.28 ± 0.62
Beta S	10.48 ± 1.05	6.97 ± 1.41	0.41 ± 0.05	0.33 ± 0.05	2.74 ± 0.94
Delta S	3.08 ± 0.31	2.07 ± 0.39	0.17 ± 0.02	0.22 ± 0.07	0.24 ± 0.08
Omicron BA.4 RBD	ND	26.7 ± 3.7	ND	19.94 ± 3.22	13.5 ± 5.5

ND: Not determined

Table S2. Summary of the cryo-EM data collection and refinement.

	RBD-Fab-AM032-0-AM047-0 (EMD-35930, PDB ID: 8J1Q)	RBD-Fab-AM032-4-AM047-6 (EMD-35945 PDB ID: 8J26)
Magnification	105,000	105,000
Voltage (kV)	300	300
Electron exposure (e/Å^2)	50	60
Defocus (μm)	-0.8 to -2.0	-0.7 to -1.6
Pixel size (Å)	0.85	0.85
Symmetry	C1	C1
Initial particles	5,448,100	1,938,408
Final particles	387,167	212,355
Map resolution (Å)	3.32Å	3.43Å
FSC threshold	0.143	0.143
Initial model (PDB)	RBD (7KGJ), Fab (6XDG)	RBD-Fab-AM032-0-AM047-0 (8J1Q)
Model resolution<br>FSC model (0.0/0.143/0.5)	3.2/3.7/4.1 (masked)<br>3.2/3.7/4.2 (unmasked)	2.9/3.2/4.0 (masked)<br>3.0/3.3/4.0 (unmasked)
non-hydrogen atoms	5447	5406
Protein residues	427	427
Nucleotides	90	90
B factor (Å^2) (min/max/mean)		
Protein	28.98/93.93/46.32	28.98/93.93/46.32
Nucleotide	20.00/79.49/34.50	20.00/79.49/34.32
r.m.s. deviations		
bond lengths (Å)	0.003 (0)	0.003 (0)

bond Angles (Å)	1.301 (4)	1.247 (3)
Molprobity score	1.9	1.89
Clashscore	8.22	7.41
Ramachandran Plot (favored/allowed/outliers %)	92.87/7.13/0.00	92.16/7.84/0.00

Table S3. Nucleotides in aptamers and amino acid residues at the interacting interfaces with a distance cut-off of 4 Å.

**AM032-0 AM032-4**	**RBD**	**AM047-0 AM047-6**	**RBD**
C32	R403	Nap-dU17	Y365
Nap-dU34	F456	G19	S366
A35	G476	Nap-dU20	L368
C36	T478	Nap-dU30	Y369
Nap-dU39	E484	G31	F374
C43	G485	G33	S375
Nap-dU44	F486	Nap-dU34	T376
G45	N487	Nap-dU35	F377
Nap-dU53	Y489		K378
	Q493		P384
	S494		N388
	Y495		G404
	G496		V407
	Y505		A435
			Y508

Table S4. Mutations in RBD observed at the binding surface of aptamers AM032-0 and AM047-0.

	**Mutations in RBD-Aptamer binding interface**	
**SARS CoV-2 Variants**	**In AM032-0 binding site**	**In AM047-0 binding site**
**Omicron B.1.1.529 [1]**	**S477N, T478K, E484A, Q493R, G496S, Q498R, N501Y, Y505H**	**S371L, S373P, S375F**
**Omicron BA.4 /5 [2]**	**S477N, T478K, E484A, F486V, Q498R, N501Y, Y505H**	**S371F, S373P, S375F, T376A**
**Delta [3]**	**T478K**	**No mutation**

Supplementary notes

Structure of AM032-0 and AM032-4

Aptamer AM032-0 consists of 80 nucleotides with 11 non-conventional nucleotides (Nap-dUs) (Figure 3a). The overall structure adopts a stem structure and a complex loop structure with three turns. Here, we refined the structure of AM032-0 spanning the residues A24 to G65 due to the high flexibility in other regions of the aptamer (Figures 2a,b). In the refined model, several non-Watson-Crick (nWC) base-parings were observed along with several base-stackings. The helical stem region (A24 to C31, Nap-dU55 to G65) contains six nWC base-pairings (A24-C63, G25-A60, Nap-dU27-G58, G28-G57, A29-A56, and A30-Nap-dU55) according to the analysis by W3DNA [4] and several base-stackings (G26-Nap-dU27, A30-C31, A56-G57, A62-C63, and C64-G65) [5]. These nWC base-pairings and base-stackings maintain the helical stem structure in AM032-0. Additionally, A60 interacts with three nucleotides (A24, C61, and A62) by hydrogen bonds, and G26 makes one hydrogen bond with G58 within this stem [6,7].

Following the stem structure of AM032-0, a large loop structure resembling a squid fin adopts three turns: T1 (from C32 to Nap-dU39), T2 (from C40 to A47), and T3 (from Nap-dU48 to G54) spanning C32 to G54. Several modified nucleotides (Nap-dUs) existing in this complex loop play a crucial role in maintaining the configuration through their hydrophobic interactions (Figures 3b,c). According to the secondary structure analysis by W3DNA [4], three nWC base-pairings (Nap-dU33-G54, Nap-dU38-G52, and G46-C49) exist within this loop structure. According to the secondary structure analysis by phenix [5], two stacking interactions (Nap-dU34-A35 and A41-A42) reside in T1 (from C32 to Nap 39) and T2 (from C40 to A47). Another stacking pair exists between G37 of T1 and G52 of T3 which seems to play an important role in holding these two turns together. Even though not many base-pairing or base-stacking exist in this complex loop, the modified Nap-dU nucleotides (Nap-dU33, 34, 38, 39, 44, 48, 51, and 53)

play a pivotal role in the stabilization of the loop through many pi-pi interactions among them and with other nucleotides (Figure 3b). Moreover, several hydrogen bonds exist between several nucleotides (A41-Nap-dU51, C43-G45, Nap-dU44-C49, and G50-Nap-dU51). Furthermore, Nap-dU33 of T1 base-pairs with G54 of T3 and also makes a contact with Nap-dU55 in the stem region, which creates a 3-residue motif (G54-Nap-dU33-Nap-dU55) at the junction of loop and stem structures.

Aptamer AM032-4 comprises of 44 nucleotides (23-66 nucleotides of AM032-0) with 10 Nap-dUs (Supplementary Figures S4a and S5h). Structurally AM032-4 is similar to AM032-0 in the loop area, but the stem region has nucleotide changes (Figures 5g,h). In the stem region base pairing of A24-C63 replaced by C63-C64 and base pairing of T27-G58 remain as like AM032-0 where Nap-dU27 replaced by T to reduce the number of Nap-dUs. A60 of the AM032-0 replaced by G60 in AM032-4 and form hydrogen bond with C61.

Structure of AM047-0 and AM047-6

The Aptamer AM047-0 is composed of 52 nucleotides with 11 Nap-dU (Figure 4a). The structure refined from G3 to C50 nucleotide regions. The first eight (the picture shows six nucleotides) nucleotides from the 5′ and 3′ ends build the first stem (Stem 1) structure with 3 Watson-Crick (WC) base-pairing (G3-C50, G6-C47, and G7-C46) of Saenger class 19 [8] and 1 nWC base-pairing (C5-G48) according to the analysis W3DNA [4] (Figure 4b). The G6-C47 base-pairing is established by only one hydrogen bond. Presence of two Nap-dU (Nap-dU4 and Nap-dU8) at this stem region hinders the WC base-pairing among all the complementary bases in this stem [9]. Nucleotides Nap-dU8 and G45 in stem 1 does not participate in base-pairing. Besides, base-stackings between adjacent nucleotides (C3-Nap-dU4, Nap-dU4-C5, C5-G6, G6-G7, G7-Nap-dU8, C47-G48, G48-A49 and A49-C50) exist in the stem 1. Following the stem 1, the nucleotides from A9 to G15 and from A40 to G45 make in short turn structures T1 and T2, respectively. In the case of T1, the base A9 points outside of helix resulting in a sharp turn (A9 to G12) and then forms a single-stranded helical turn through base-stackings (G13-A14, A14-G15, and G15-C16). After T1, loop 1 (L1, from C16 to A23) is formed with 3 Nap-dUs (Nap-dU17, Nap-dU20, and Nap-dU22). Nap-dU17 makes an nWC base-pair with G36, and Nap-dU22 interacts with Nap-dU41 through pi-pi interaction (Figure 4b). The L1 is connected with loop 2 (L2) that is composed of 6 nucleotides (Nap-dU30 to Nap-dU35) with 3 Nap-dUs at 30, 34, and 35 (Figure 4c). Nap-dU34 points inside the loop and makes pi interactions with G31, A32, and G37 of S2. Nap-dU30 and Nap-dU35 make nWC base-pairings with G37 and G31, respectively. The Nap-dU30 and 35 make pi-pi interactions with Nap-dU17 and 20 of L1, which contribute to a stable configuration between loops 1 and 2 (Figures 4b, c). The L2 is followed by a short stem (stem 2) that is composed of C26 to C29 and G36 to G39. The stem 2 (S2) is formed by 1 WC base-pairing of Saenger class 19 (C28-G39) and 2 nWC base-pairings (C26-A40, C29-G38). The S2 is further stabilized by base-stackings of G25-C26, C28-C29, G38-G39, and G39-A40. C26 and G37 make hydrogen bonds with G15 of T1 and Nap-dU30 of L2, respectively. Interestingly, the stem 2 doesn’t adopt a standard form of DNA helix. Instead, it seems to have plasticity to make two stable loops L1 and L2. In the case of T2, G45 and C44 have a base-stacking, and Nap-dU43 has a pi-pi interaction with Nap-dU24. And Nap-dU41 interacts with A40 and Nap-dU22 of L1.

The optimized aptamer AM047-6 is composed of 48 nucleotides (G3 to C50 of AM032-0) with 8 Nap-dUs (Supplementary Figures S4b and S5i). The AM047-6 maintains the same overall structure as AM047-0 (Figures 5g,i) but has reduced size. The major changes exist in the S1 region where Nap-dU4, Nap-dU8, A45, and A49 were changed to G4, C8, G45, and C49 to stabilize the S1, reduce the size, and reduce the number of Nap-dUs. WC base-pairings (G3-C50, G4-C49, C5-G48, G6-C47, G7-C46) and base-stackings (C46-C47, G48-C49, and C49-C50) are observed in the cryo-EM structure of AM047-6 (Figures 5g,i).

Aptamer model building using auto-DRRAFTER

The auto-DRRAFTER [10] is designed to build RNA models using cryo-EM maps. However, we used this software to build ssDNA aptamers models since ssDNAs are flexible and form base pairs like RNA molecules [11]. Briefly, the secondary structures of aptamers were roughly predicted by mFold [12] considering the DNA sequences as RNA sequences. And the primary sequences of aptamers, mFold-predicted secondary structures, and the cryo-EM density maps were used to generate 3D models by auto-DRRATER [10]. One of the helix segments, which was predicted by auto-DRRAFTER [10] at the initial setting step, was selected and the helix structure was adjusted to the cryo-EM density maps using Chimera program [7]. The adjusted PDB file generated by Chimera was used in setting auto-DRRAFTER program manually and the first round of auto-DRRAFTER was run to generate 3D models. The top ten scoring models were selected, and the highly convergent helical structures were adjusted to fit with cryo-EM density maps using the Chimera [7]. The second round of auto-DRRAFTER was executed using the adjusted PDB file. We repeated one or two more rounds of auto-DRRAFTER analyses. Finally, the best model acquired from the last round was used for further coordination into the cryo-EM density map through refinements with Coot [13] and Phenix [5].

References:

1. Chakraborty, C.; Bhattacharya, M.; Sharma, A.R.; Mallik, B. Omicron (B.1.1.529) - A new heavily mutated variant: Mapped location and probable properties of its mutations with an emphasis on S-glycoprotein. *Int J Biol Macromol* **2022**, *219*, 980-997. doi:10.1016/j.ijbiomac.2022.07.254.
2. Tegally, H.; Moir, M.; Everatt, J.; Giovanetti, M.; Scheepers, C.; Wilkinson, E.; Subramoney, K.; Makatini, Z.; Moyo, S.; Amoako, D.G.; et al. Emergence of SARS-CoV-2 Omicron lineages BA.4 and BA.5 in South Africa. *Nat Med* **2022**, *28*, 1785-1790. doi:10.1038/s41591-022-01911-2.
3. Dhawan, M.; Sharma, A.; Priyanka; Thakur, N.; Rajkhowa, T.K.; Choudhary, O.P. Delta variant (B.1.617.2) of SARS-CoV-2: Mutations, impact, challenges and possible solutions. *Hum Vacc Immunother* **2022**, *18*. doi:10.1080/21645515.2022.2068883.
4. Li, S.X.; Olson, W.K.; Lu, X.J. Web 3DNA 2.0 for the analysis, visualization, and modeling of 3D nucleic acid structures. *Nucleic Acids Research* **2019**, *47*, W26-W34. doi:10.1093/nar/gkz394.
5. Liebschner, D.; Afonine, P.V.; Baker, M.L.; Bunkoczi, G.; Chen, V.B.; Croll, T.I.; Hintze, B.; Hung, L.W.; Jain, S.; McCoy, A.J.; et al. Macromolecular structure determination using X-rays, neutrons and electrons: recent developments in Phenix. *Acta Crystallogr D Struct Biol* **2019**, *75*, 861-877. doi:10.1107/S2059798319011471.

6. Mills, J.E.J.; Dean, P.M. Three-dimensional hydrogen-bond geometry and probability information from a crystal survey. *J Comput Aid Mol Des* **1996**, *10*, 607-622. doi:Doi 10.1007/Bf00134183.
7. Pettersen, E.F.; Goddard, T.D.; Huang, C.C.; Couch, G.S.; Greenblatt, D.M.; Meng, E.C.; Ferrin, T.E. UCSF chimera - A visualization system for exploratory research and analysis. *J Comput Chem* **2004**, *25*, 1605-1612. doi:10.1002/jcc.20084.
8. Saenger, W. *Principles of Nucliec Acid Structure*; Springer-Verlag New York Inc.: New York, 1984; pp. 120-121.
9. Wang, D.L.; Zhang, S.Y.; Li, L.A.; Liu, X.; Mei, K.R.; Wang, X.Q. Structural insights into the assembly and activation of IL-1 beta with its receptors. *Nat Immunol* **2010**, *11*, 905-U952. doi:10.1038/ni.1925.
10. Kappel, K.; Zhang, K.; Su, Z.; Watkins, A.M.; Kladwang, W.; Li, S.; Pintilie, G.; Topkar, V.V.; Rangan, R.; Zheludev, I.N.; et al. Accelerated cryo-EM-guided determination of three-dimensional RNA-only structures. *Nat. Methods* **2020**, *17*, 699-707. doi:10.1038/s41592-020-0878-9.
11. Bao, L.; Zhang, X.; Jin, L.; Tan, Z.J. Flexibility of nucleic acids: From DNA to RNA. *Chinese Phys B* **2016**, *25*, doi:10.1088/1674-1056/25/1/018703
12. M. Zuker. Mfold web server for nucleic acid folding and hybridization prediction. *Nucleic Acids Res* **2003**. *31 (13)*, 3406-15
13. Emsley, P.; Cowtan, K. Coot: model-building tools for molecular graphics. *Acta Crystallogr. D* **2004**, *60*, 2126-2132. doi:10.1107/S0907444904019158.
